# Spectroscopic Properties of Two 5′-(4-Dimethylamino)Azobenzene Conjugated G-Quadruplex Forming Oligonucleotides

**DOI:** 10.3390/ijms21197103

**Published:** 2020-09-26

**Authors:** Concetta Imperatore, Antonio Varriale, Elisa Rivieccio, Angela Pennacchio, Maria Staiano, Sabato D’Auria, Marcello Casertano, Carlo Altucci, Mohammadhassan Valadan, Manjot Singh, Marialuisa Menna, Michela Varra

**Affiliations:** 1 Department of Pharmacy, School of Medicine, University of Naples “Federico II”, Via D. Montesano 49, 80131 Naples, Italy; cimperat@unina.it (C.I.); elisrivi90@gmail.com (E.R.); marcello.casertano@unina.it (M.C.); mlmenna@unina.it (M.M.); 2 Institute of Food Sciences, National Research Council of Italy, via Roma 64, 83100 Avellino, Italy; antonio.varriale@isa.cnr.it (A.V.); angela.pennacchio@isa.cnr.it (A.P.); maria.staiano@isa.cnr.it (M.S.); sabato.dauria@cnr.it (S.D.); 3 Department of Physics “Ettore Pancini”, University of Naples Federico II, Via Cinthia, 21—Building 6, 80126 Naples, Italy; altucci@na.infn.it (C.A.); mohammadhassan.valadan@unina.it (M.V.); manjot.singh@unina.it (M.S.)

**Keywords:** G-quadruplexes, conjugated aptamers, CD G-quadruplexes, fluorescence G-quadruplexes, azobenzenes, trans-cis conversion, 5′-(4-dimethylamino)azobenzene phosphoramidite derivative

## Abstract

The synthesis of two 5′-end (4-dimethylamino)azobenzene conjugated G-quadruplex forming aptamers, the thrombin binding aptamer (TBA) and the HIV-1 integrase aptamer (T30695), was performed. Their structural behavior was investigated by means of UV, CD, fluorescence spectroscopy, and gel electrophoresis techniques in K^+^-containing buffers and water-ethanol blends. Particularly, we observed that the presence of the 5′-(4-dimethylamino)azobenzene moiety leads TBA to form multimers instead of the typical monomolecular chair-like G-quadruplex and almost hampers T30695 G-quadruplex monomers to dimerize. Fluorescence studies evidenced that both the conjugated G-quadruplexes possess unique fluorescence features when excited at wavelengths corresponding to the UV absorption of the conjugated moiety. Furthermore, a preliminary investigation of the trans-cis conversion of the dye incorporated at the 5′-end of TBA and T30695 showed that, unlike the free dye, in K^+^-containing water-ethanol-triethylamine blend the trans-to-cis conversion was almost undetectable by means of a standard UV spectrophotometer.

## 1. Introduction

Currently, the higher-order noncanonical G-quadruplex structures (Qs) arising from the reverse Hoogsteen-like pairing of four guanines (G-tetrad) are the most frequently studied DNA structures. Their involvement in important biological functions, such as telomere maintenance [1,2,3], DNA-protein recognition [4,5] and protein inhibition [6,7] has been supposed. Moreover, the stability of Qs in a large variety of experimental conditions has encouraged their exploitation in biotechnology, affording a reliable scaffold for biosensors and nanomechanical devices [8,9,10] where they can also form hybrid structures with novel two-dimensional materials [11,12].

Qs are often modified with suitable molecules in order to tune their stability or to provide them with specific properties such as fluorescence, chemical resistance, etc. [13,14,15,16]. Among the great number of molecules potentially appropriate for oligonucleotide (ON) modification, azobenzene and its derivatives, characterized by an extensive aromatic core, are of particular interest. Their introduction into the ON structure, indeed, has the advantage of producing end-capping photoswitches, a type of derivative whose structure and/or function can be modulated with a high spatial and temporal resolution by using light at a specific wavelength [17].

Many studies have been performed on the azobenzene linked to ONs forming duplexes or hairpins [17,18,19]; in contrast, few examples of Qs incorporating azobenzene have been reported. Thevarpadam et al. developed a minimal light-switchable DNA module enabling the formation of an intermolecular Q [20], and Mo et al. have replaced the thrombin binding aptamer (TBA) loop with a photoactivatable azobenzene residue controlling the thrombin enzyme activity [21]. In both cases, a 4,4′-bis(hydroxymethyl)azobenzene moiety was used as the scaffold to produce appropriate derivatives useful for the modification of Qs through a backbone inclusion approach, with the azobenzene moiety acting as a loop of the folded Q. To the best of our knowledge, no end capped azobenzene G-rich oligonucleotide (GRO) has been reported to date. However, the exploration of the structural properties of 5′-azobenzene conjugated Q-forming aptamers may be the crucial issue for their potential use both in the regulation of the biomolecular processes in which aptamers are involved and in the design of innovative nanotechnological applications (e.g., biosensors and drug delivery) [22,23].

**Figure 1:**
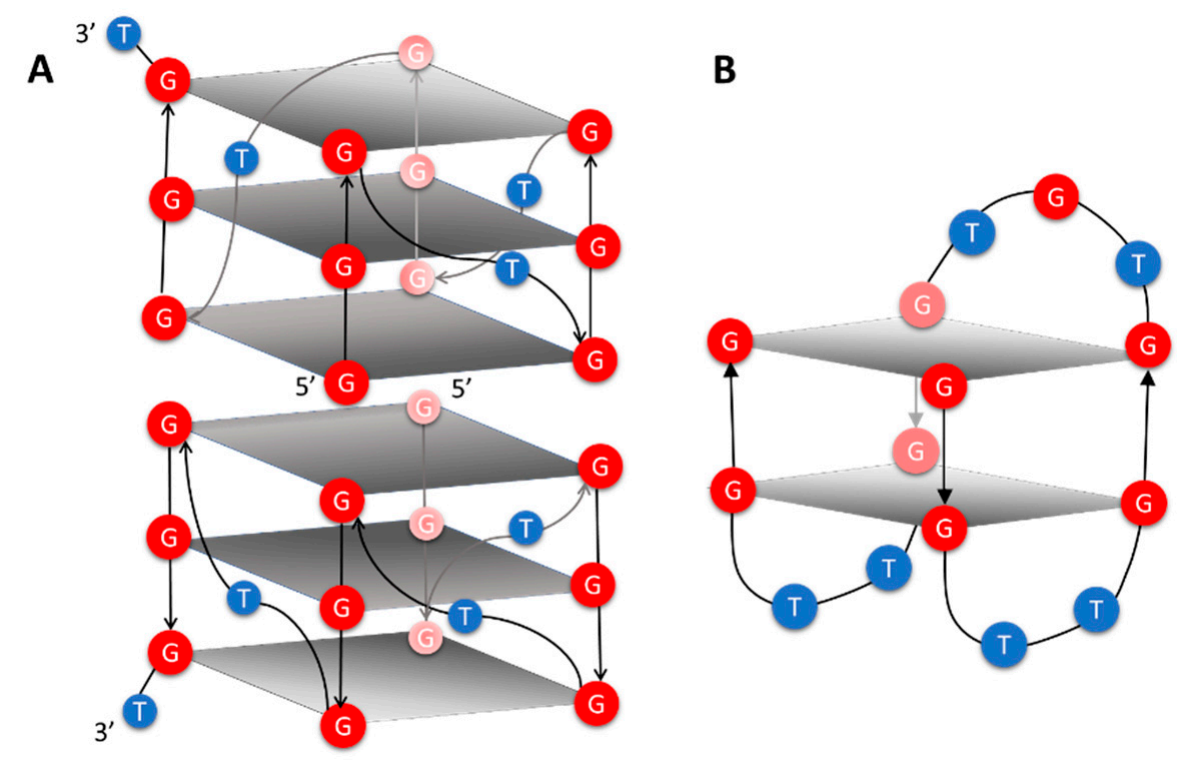
Schematic representation of T30695 (**A**) and TBA (**B**) Q structures. Red and blue balls containing the letters G and T represent G and T residues, respectively. Gray rectangles schematize the G-quartets, regardless the syn-anti orientation of each G residue.

On this basis, we started to explore the structural properties of DNA Qs formed by 5′-(4-dimethylamino)azobenzene conjugating two well-known Q forming aptamers, T30695 (5′GGGTGGGTGGGTGGGT3′) and TBA (5′GGTTGGTGTGGTTGG3′). T30695 folds in very stable Q dimers by means of the stacking of two parallel-stranded monomeric subunits (Figure 1A) at their 5′-end [24]. In each monomer, three double chain-reversal loops, formed by a single T residue, laterally connect the four G_3_-tracts. TBA folds into a chair-like monomolecular Q (Figure 1B) having one TGT and two TT lateral loops that connect the four G_2_ tracts. Some nucleobases from residues forming the loops are involved in stacking interactions with both 5′- and 3′-end G-quartets. In particular, G_8_ and T_9_ in the T_7_G_8_T_9_ loop stack on the G-quartet at the 5′-end whereas T_4_ and T_13_, in the T_12_T_13_ and T_3_T_4_ loops, stack on the G-quartet at the 3′-end. Structural studies, performed by means of both X-ray and NMR techniques, showed that T_4_, G_8_, T_9_, and T_13_ have an important role in stabilizing the TBA-Q [25,26,27,28].

Therefore, the G-quartet at the 5′-end makes important but very different stacking interactions in T30695- and TBA-Qs. Indeed, in T30695-Q two G-quartets at the 5′-ends of the two monomers form the center of the Q dimer interacting with each other and with the adjacent G-quartets, whereas the G-quartet at the 5′-end of TBA-Q is sandwiched between the G-quartet at the 3′-end and G_8_T_9_. Another important difference between the two Qs relates to the orientation of the G nucleobase with respect to 2′-deoxy-ribose moiety, deriving from the rotation around the *N*-glycosidic bond. Indeed, all the G residues forming T30605-Qs are in an anti orientation, whereas the G residues forming each G-quartet of TBA-Q alternate between syn and anti orientations.

Herein we report the synthesis of a conjugated 5′-(4-dimethylamino)azobenzene TBA and T30695 and provide an evaluation of the effects of the conjugation on the ability of each sequence to fold in the corresponding Q. To achieve this purpose, an extensive UV, CD, fluorescence, and gel electrophoresis analysis has been carried out on the resulting Q variants and, for comparison, on the unmodified Qs. We have also monitored the cis-trans photoconversion under different solvent conditions of the free 4-(dimethylamino)azobenzene derivative and the corresponding end capping TBA and T30695 dye by standard UV methods in order to assess their photoresponsive properties. In particular, we have replaced the common buffered solutions used for the analyses of the structured nucleic acids with H_2_O: EtOH blends due to the poor solubility of the free 4-(dimethylamino)azobenzene derivative.

Ultimately, the whole set of our results constitutes a solid basis of chemical and physical knowledge about *(i)* the Q-folding capacity of the two sequences T30695 and TBA in comparison with those of the corresponding (4-dimethylamino)azobenzene conjugated ones, about *(ii)* the structural behavior of the unmodified Qs and their variants in solution with different solvents and, finally, on *(iii)* their photoresponsive properties. This information may be useful in any future biological and biotechnological application envisaged for these two aptamers, taking into account that both T30695 and TBA have an important biological activity, since T30695 binds to and inhibits the HIV-1 integrase at nanomolar concentrations [24,29,30], whereas TBA [27,28,31,32,33,34] binds to and inhibits the thrombin, causing an anticoagulant effect.

## 2. Results

### 2.1. Chemistry

**Scheme 1:**
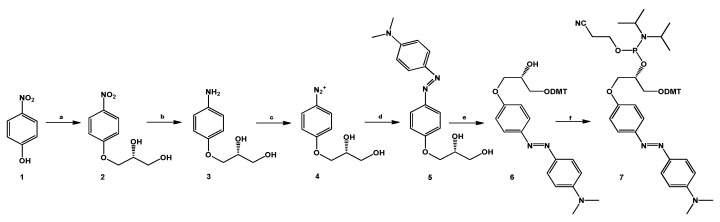
Synthesis of the DMAzo moiety. (**a**) **1**, (1.0 g, 7.2 mmol), KOH (2 mg, 3.6 × 10^−2^ mmol), R-glycidol 574 µL (0.640 g, 8.6 mmol) in dry toluene (4.0 mL) at 90 °C; (**b**) **2** (0. 560 g, 2.6 mmol), Pd/C (28 mg, 5% w/w with respect to 2) and EtOH (25 mL); (**c**) **3** (0.400 g, 2.2 mmol), 205 µL fluoroboric acid (water solution, 48%, 3.3 mmol), EtOH (4.0 mL), −15 °C, isoamyl nitrite (0.258 g, 2.2 mmol); (**d**) **4** (0.360 g, 1.8 mmol) acetic acid (10 mL, 14.0 g, 232 mmol), sodium acetate (4.0 g, 511 mmol) and *N*,*N*-dimethylaniline (254 µL, 0.270 g, 2.2 mmol). (**e**) **5** (0.400 g, 1.3 mmol), 4,4-dimethoxytrityl chloride (0.430 g, 1.3 mmol), 4-dimethylaminopyridine (0.0076 g, 0.062 mmol) dry Py (6.35 mL), 2.5 h and (**f**) **6** (0.350 g, 0.57 mmol), *β*-cyanoethyl-*N*,*N*’-diisopropyl-chloro-phosphoramidite (0.190 mL, 0.85 mmol), DIPEA (0.296 mL, 1.7 mmol), dry DCM (3 mL), 1.0 h.

The (*R*) 4-(dimethylamino)azobenzene glycerol derivative (DMAzo, 5, Scheme 1) was realized according to our recently reported synthetic route adopted for the synthesis of some pyrrole azobenzenes [35,36]. The two hydroxyl groups of the glycerol linker inserted on one benzene ring are used to convert the azobenzene derivative into an appropriate phosphoramidite building block by using the classic procedure (see the Materials and Methods section) [16]. The stability of the new phosphoramidite found in each cycle of the automated solid-phase synthesis under standard chemical conditions was verified before its incorporation at the 5′-end of the two selected aptamer sequences, executing a coupling step with standard solid supports in CPG (controlled pore glass) functionalized with G or T. T30695 and TBA were first synthesized by automated solid phase synthesis and, still anchored to the solid support, coupled manually with compound **7** using two coupling cycles (2 × 20 min).

### 2.2. UV and CD Spectroscopy

**Figure 2:**
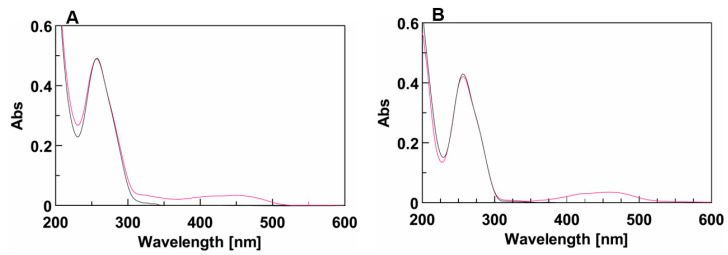
(**A**) UV spectra of TBA (black line) and 5′-DMAzo-TBA (fuchsia line) in 100 K buffer (pH = 7.4) in the range 200–600 nm. (**B**) UV spectra of T30695 (black line) and 5′-DMAzo-T30695 (fuchsia line) in 10 K buffer (pH = 7.4), in the range 200–600 nm. The spectra were acquired at 10 °C using [ON] = 20 µM. cuvette o.l. 0.1 cm, V = 400 µL.

The effect of the DMAzo conjugation on the TBA and T30695 Qs, was explored by means of UV and CD spectroscopy. The experiments were performed using different solvent conditions. Potassium ions are commonly added to solutions to stabilize Q structures. The folding into a stable TBA-Q requires a high concentration of potassium ions, usually 100 mM [32,33,37], whereas, due to the strong stability of T30695-Q, the achievement of the corresponding CD melting curves in the selected temperature range (10–100 °C) requires the reduction of the potassium ion concentration to 10 mM [24,29,30]. On this basis, we used 10 mM of phosphate buffer containing 90 mM of KCl (100 K buffer, pH = 7.4) for the TBA and 5′-DMAzo-TBA and 10 mM of potassium phosphate buffer (10 K buffer, pH = 7.4) for the T30695 and 5′-DMAzo-T30695. As mentioned above, all the sequences were also examined using a mixture of EtOH: H_2_O 1:1 v/v containing 5 mM of KOH (5KEW). In all the explored conditions, in addition to the typical UV band at about 260 nm due to the absorption of the ONs, the UV profiles of 5′-DMAzo-TBA and 5′-DMAzo-T30695 contained a broad band at about 450 nm, attributable to the presence of the DMAzo moiety at the 5′-end of the two ONs (Figure 2, Supplementary Figures S1 and S2).

**Figure 3:**
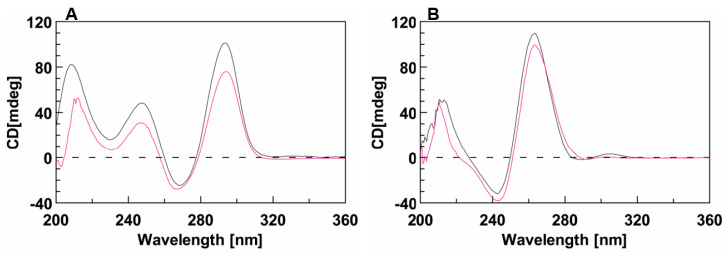
CD spectra of (**A**) TBA (black line) and 5′-DMAzo-TBA (fuchsia line), acquired in 100 K buffer (pH = 7.4) and of (**B**) T30695 (black line) and 5′-DMAzo-T30695 (fuchsia line), acquired in 10 K buffer (pH = 7.4). T = 10 °C; [ON] = 20 µM. Cuvette o.l. 1.0 cm, Vol = 1400 µL.

Circular dichroism (CD) analyses performed on both the unmodified and conjugated sequences showed that the presence of the DMAzo moiety did not significantly affect the CD profiles of the two aptamers. Indeed, similarly to the unmodified TBA (Figure 3A, black line), the CD profile of 5′-DMAzo-TBA is characterized by three positive bands at 212, 247, and 295 nm and a negative one around 270 nm. These bands can be attributed to the presence of an antiparallel Q in the given solutions (Figure 3A fuchsia line) [32,33,37,38]. In 10 K buffer, the CD profiles of T30695 (Figure 3B black line) and of 5′-DMAzo-T30695 (Figure 3B, fuchsia line) are both characterized by two positive CD bands at about 209 and 262 nm and a negative one at 242 nm, thus suggesting that the conjugated sequence preserved the same ability as T30695 to fold into parallel Qs. A weak positive CD band around 300 nm appeared only in the CD profile of the unmodified T30695. Significantly, in the CD profiles of both conjugated ONs, there were no significant bands correlated with the UV band at about 450 nm.

A similar behavior was observed using the 5KEW blend, with the exception of slight redshifts of the CD bands of 5′-DMAzo-T30695 (Supplementary Figure S3B′).

**Figure 4:**
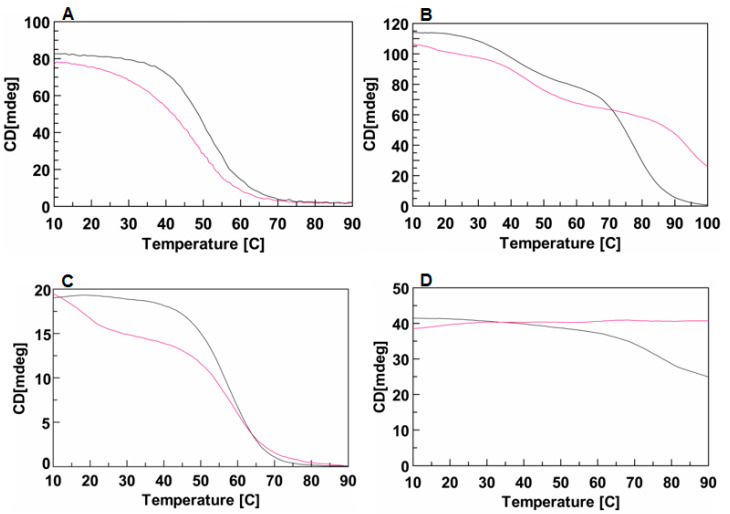
CD melting profiles of TBA or T30695 and of the corresponding conjugated sequences in different solvent conditions. (**A**) TBA (black line) and 5′-DMAzo-TBA (fuchsia line) in 100 K buffer (pH=7.4). (**B**) T30695 (black line) and 5′-DMAzo-T30695 (fuchsia line), in 10 K buffer (pH = 7.4). (**C**) TBA (black line) and 5′-DMAzo-TBA (fuchsia line) in 5KEW blend. (**D**) T30695 (black line) and 5′-DMAzo-T30695 (fuchsia line) in 5KEW blend. [ON] = 2.0 µM.

In order to quantify the effect of the DMAzo conjugation on the stability of the TBA- and T30695-Qs, CD melting experiments were performed. Figure 4A shows the CD melting curves of TBA (black) and of 5′-DMAzo-TBA (fuchsia), in the 100 K buffer, carried out by monitoring the amplitude of the CD band at 295 nm, increasing the temperature from 10 to 90 °C.

The apparent melting temperature, evaluated from the minimum in the first derivative of each melting curve, suggested that the conjugation with the DMAzo moiety significantly reduced the thermal stability of the TBA Q structure (ΔT_m_∼−6 °C).

According to literature data, the CD melting curve of T30695 shows two inflection points accounting for two unfolding events, 5′-5′ end-to-end stacked dimeric Qs → Qs monomers → unfolded single strands [24,29,30]. The melting curve of 5′-DMAzo-T30695 was very similar to that of T30695, except for the second inflection point, which occurred at T higher than 90 °C (Figure 4B). Indeed, the monitored CD band appeared to persist also at 100 °C suggesting that the source of this CD signal was still in solution at the highest explored temperature whereas the CD band at 263 nm caused by T30695-Q almost completely disappeared at T ~90 °C. The data obtained strongly suggested that the conjugation of the DMAzo at the 5′-end of T30695-Q caused a strong stabilizing effect on the monomolecular T30695-Q.

In agreement with the CD melting results in 100 K buffer, in the 5KEW blend, the introduction of DMAzo at the 5′-end of TBA also appeared to negatively affect the stability of the folded Q(s) (Figure 4C), while the 5′-DMAzo-T30695 seemed not to be affected by increasing the temperature up to 90 °C (Figure 4D, fuchsia line). In the adopted condition, T30695-Q(s) also appeared to melt only partially (Figure 4D, black line). Therefore, unlike for TBA, the T30695-Q(s) appeared to be stabilized by introducing the DMAzo moiety at the 5′-end of the sequence. The CD spectra at different temperatures in the range 20–60 °C for TBA and 5′-DMAzo-TBA and 20–90 °C for T30695 and 5′-DMAzo-T30695 were also acquired using the 5KEW blend (Supplementary Figures S4 and S5A–D). As expected, with the increase of the temperature from 20 to 60 °C the amplitude of the CD band at around 290 nm produced by the 5′-DMAzo-TBA-Q(s) decreased more rapidly than that of TBA (Figure S5A,B). In agreement with the CD melting results, the CD profiles of 5′-DMAzo-T30695 did not show any significant changes, whereas the positive CD band at 263 nm produced by T30695 gradually decreased (by about 30%) with an increase of the temperature from 20 to 90 °C (Supplementary Figures S4 and S5C,D).

### 2.3. Fluorescence Results

**Figure 5:**
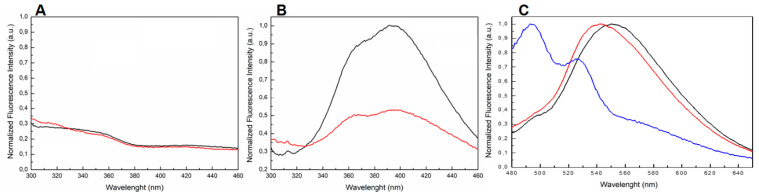
The normalized fluorescence spectra of (**A**) TBA (black line) and 5′-DMAzo-TBA (red line) in 100 K buffer excited at 255 nm and acquired in the range 300–460 nm. (**B**) T30695 (black line) and 5′-DMAzo-T30695 (red line) in 10 K buffer excited at 255 nm and acquired in the range 300–460 nm. (**C**) 5′-DMAzo-TBA (100 K buffer, red line), 5′-DMAzo-T30695 (10K buffer, black line) and free DMAzo (10 K buffer, blue line) excited at 450 nm and acquired in the range 480–650 nm.

It is well known that Qs produce a fluorescent emission band around 400 nm when excited with wavelength in the range 255–265 nm [39,40,41,42,43,44,45,46,47,48,49]. Our fluorescence experiments revealed that both TBA and 5′-DMAzo-TBA, annealed in 100 K buffer were unable to produce a significant emission band in the range 300–460 nm (Figure 5A, black and red lines respectively). In agreement with the literature data [47,48,49], the fluorescence spectra of T30695 excited at 255 nm showed two broad bands in the wavelength range 300–460 nm (Figure 5B, black line). However, the conjugation of T30695 at the 5′-end with the DMAzo moiety strongly reduced this emission (Figure 5B, red line). Moreover, the UV spectra of both 5′-DMAzo-TBA and 5′-DMAzo-T30695 also showed an additional UV band around 450 nm (Figure 2). We also excited the two conjugated ONs at 450 nm, acquiring the steady-state emission spectra in the range of 480–650 nm. The fluorescence results showed emissions centered at 560 nm (Figure 5C and Supplementary Figure S6, black and red lines), which are very different both in their profile and intensity from those of the free DMAzo moiety (Figure 5C and Supplementary Figure S6, blue line). It is noteworthy that, in both cases, an increase in the fluorescence of about seven-fold was evidenced.

**Figure 6:**
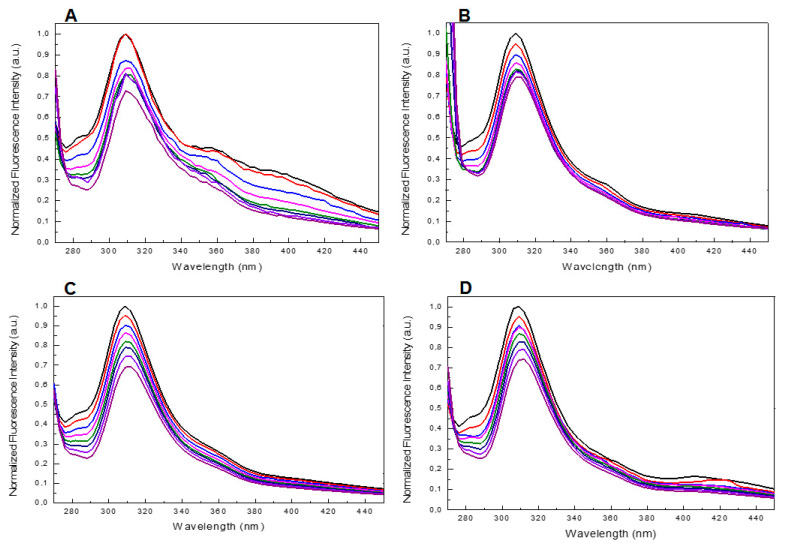
(**A**,**B**) Fluorescence spectra at different temperatures (from 25 to 95 °C, ΔT = 10 °C) of T30695 and 5′-DMAzo-T30695 excited at 255 nm acquired in the region 300–460 nm. (**C**,**D**). Fluorescence spectra at different temperatures (from 25 to 95 °C, ΔT = 10 °C) of TBA and 5′-DMAzo-TBA excited at 255 nm acquired in the region 300–460 nm. In all cases 5KWE was used as a solvent.

**Figure 7:**
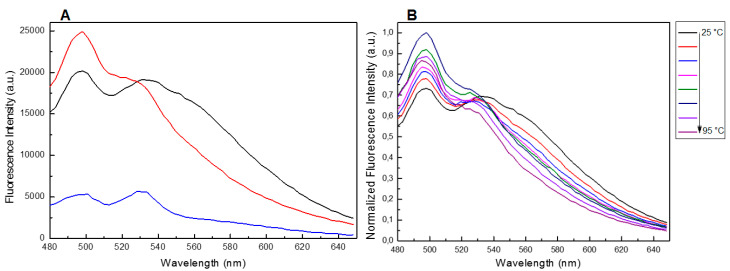
(**A**) Fluorescence spectra of the free DMAzo (blue line), 5′-DMAzo-TBA (red line) and 5′-DMAzo-T30695 (black line) at 25 °C in the wavelength range 480–650 nm, after excitation at 450 nm. (**B**) Temperature-dependent fluorescence spectra of the 5′-DMAzo-T30695, in the wavelength range 480–650 nm, after excitation at 450 nm. In all cases 5KWE was used as solvent.

Despite the fact that the UV and CD profiles of both the conjugated and unmodified ONs annealed in 5KEW (Supplementary Figures S5 and S1) were very similar to those obtained in the phosphate buffer solutions (Figure 2 and Figure 3 and Supplementary Figure S3) important differences in the steady-state emission spectra of the ONs were revealed depending on the solvent system used. Indeed, a new band centered at about 310 nm appeared after excitation at 255 nm for all species (Figure 6 and Supplementary Figure S7). Furthermore, in the case of T30695 this band was also accompanied by two other broad fluorescence emissions at about 360 and 400 nm (Figure 6A and Supplementary Figure S7). The fluorescence spectra were also acquired at different temperatures, from 25 to 95 °C (ΔT = 10 °C), after excitation at 255 nm. The fluorescence intensity of the bands produced by T30695 in the range 340–460 nm decreased with an increase in the temperature, thus suggesting that the presence of Qs in the solutions could affect the above-mentioned emissions. The fluorescence properties of the conjugated dye were further explored by the excitation of the samples at 450 nm, acquiring the fluorescence emission in the wavelength range 480–650 nm (Figure 7A). Similar to the results obtained using phosphate buffer, in 5KEW, the DMAzo conjugating ONs also emitted more intensely than the free DMAzo (Figure 5C and Supplementary Figure S6). Moreover, significant differences in the maxima of the emission bands were also revealed between the two conjugated ONs. At 25 °C the fluorescence profiles of 5′-DMAzo-TBA showed the main band centered at 397 nm and a shoulder at 530 nm (Figure 7A, red line) whereas those of the free DMAzo (Figure 7A, blue line) and 5′-DMAzo-T30695 (Figure 7A, black line) showed two bands having the same intensity at 397 and 530 nm. A fluorescence broadening in the wavelength range of 540 nm up to the fixed end-scale was observed in the case of the 5′-DMAzo-T30695. In order to acquire information about the behavior of the emitted fluorescence from the free DMAzo and the DMAzo conjugating Qs, temperature-dependent fluorescence experiments were also carried out by exciting the samples in 5KEW at 450 nm (Figure 7B, Supplementary Figures S8 and S9). The intensity of fluorescence emission of the 5′-DMAzo-TBA and of the free DMAzo (Supplementary Figure S8) decreased in the entire fixed spectral range as the temperature was increased. In the case of 5′-DMAzo-T30695, a decrease in the intensity of the fluorescence at 530 nm and a simultaneous increase at 500 nm caused an isofluorescent point at about 525 nm in the range 25–75 °C (Figure 7B and Supplementary Figure S9). These data suggest that a transition between two differently emitting states involving the conjugating ON dye followed the increase of the temperature from 10 to 75 °C. Over 75 °C, the fluorescence behavior of the 5′-DMAzo-T30695 became similar to that of the 5′-DMAzo-TBA (Supplementary Figures S8 and S9), and the increase in the temperature only provoked a decrease in the fluorescence intensity.

### 2.4. Native PAGE

**Figure 8:**
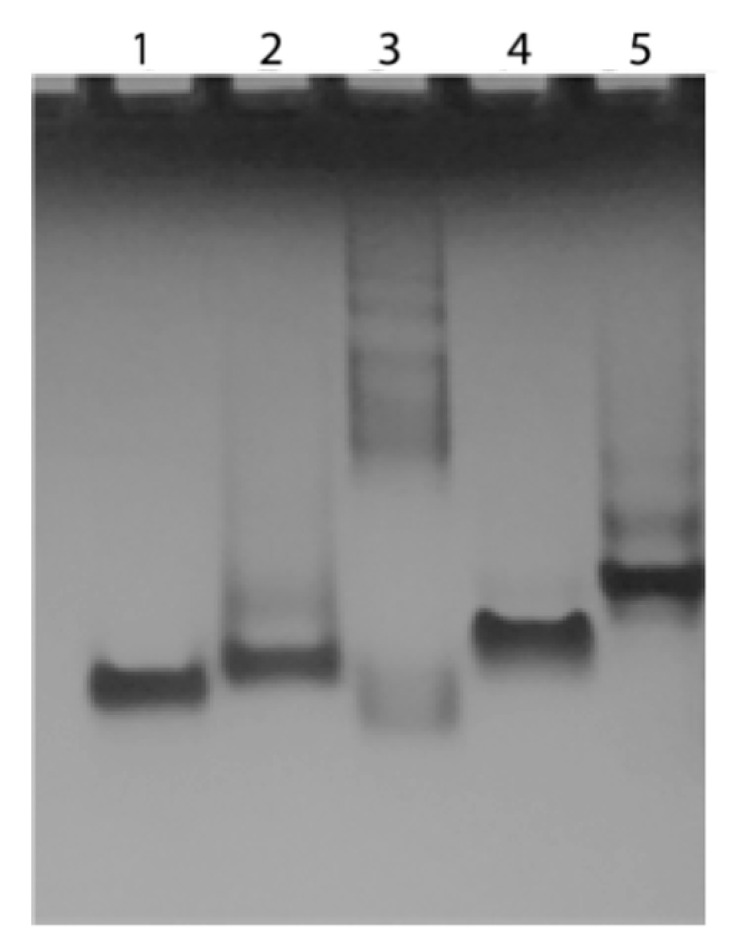
Electrophoresis mobility shift assay of the conjugated and unmodified sequences. Lane 1: TBA; lane 2: 5′-TT-T30695; lane 3: 5′-DMAzo-TBA; lane 4: 5′-DMAzo-T30695 and lane 5: T30695.

In order to verify the ability of the conjugated TBA and T30695 to fold into the corresponding Q, in the presence of K^+^ ions, a non-denaturing gel electrophoresis mobility shift assay was performed. Figure 8 shows the electrophoretic mobilities of the TBA and T30695 (lane 1 and 5, respectively), of the conjugated sequences 5′-DMAzo-TBA and 5′-DMAzo-T30695 (lanes 3 and 4), and of 5’TT-T30695 (lane 2), on cooled (8–10 °C), non-denaturing polyacrylamide gel containing 30 mM of KCl. The TBA and 5’TT-T30695 were used as internal references for the monomolecular Qs. As expected, the migrations of the TBA and 5’TT-T30695 [24,29,30,35] were faster than the main band of T30695, thus confirming that the folding of T30695 gives rise to dimeric Q. Besides the fastest main band, T30695 and 5′-TT-T30695 also show other electrophoretic bands; this phenomenon had already been observed and attributed to the formation of another type of stacked dimer (i.e., 3′-3′-end-to-end and/or 5′-3′-end-to-end stacked Q dimers) [24,29,30,35]. The 5′-DMAzo-T30695 (lane 5) migrated faster than the unmodified T30695, but slower than 5’TT-T30695, thus suggesting that the presence of the DMAzo moiety at the 5′-end modifies, at least in part, the ability of T30695 to form dimeric Qs [24,29,30,35]. Surprisingly, many slow bands are present in lane 3, thus indicating that the 5′-DMAzo-TBA forms multimer species having high molecular weights. Importantly, similar results were also obtained by annealing the ONs in 5KEW and loading the samples on gel containing 5.0 mM of KCl (Supplementary Figure S10).

### 2.5. Spectroscopic Characterization of the Free and Conjugated DMAzo

Light-activated cis isomers of DMAzo analogues generally have very short half-lives in a wide range of experimental conditions caused by acid-base equilibria involving the protonation of the diazogroup [50,51,52,53,54,55]. However, in a mildly alkaline medium, irradiation with visible light causes the trans to cis conversion of the DMAzo moieties incorporated within DNA double helix structures [17,56,57] triggering significant changes in the stability of the duplex structures. Based on these results, with the aim of verifying the effect of light irradiation on 5′-DMAzo conjugated ONs, we first explored the trans-cis conversion of free DMAzo (**5**, Scheme 1) in different water-based blends. Specifically, we performed a preliminary study on **5** in H_2_O: EtOH 50:50 containing 5 mM of KCl, varying the pH of the solution by the addition of triethylamine (TEA). The choice of these operating conditions was made according to certain important factors, namely the ability of G-rich strands to fold into Q(s) in H_2_O: EtOH blends [58,59,60], thus allowing us to avoid using buffered solutions, such as **5** which has limited solubility (<10 µM).

**Figure 9:**
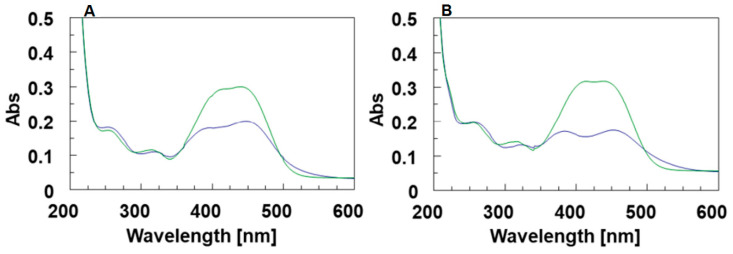
(**A**) UV spectra of the free DMAzo in the 5KEW containing 0.1 mM of TEA before (green line) and after (blue line) excitation with LED at 436 nm. (**B**) UV spectra of the free DMAzo in the 5KEW containing 0.5 mM of TEA before (green line) and after (blue line) excitation with LED at 436 nm.

First, the UV spectra of **5** (20 µM) dissolved in 5KEW before and after the irradiation with LED at 436 nm were monitored. After irradiation, the spectra did not show any change attributable to the presence of the **5**-cis isomer in the solution (Supplementary Figure S11A), thus suggesting that the cis to trans conversion could not be revealed by standard UV spectroscopy [36,51,52,53,54,55]. Similar results were also obtained with the addition of TEA at a final concentration <0.05 mM. With an increase of TEA up to 0.1 mM, the UV absorption bands of **5** maintained in the dark did not significantly change (Supplementary Figure S11B). However, after the LED irradiation the intensity of the absorption bands of **5** around 412 and 445 nm significantly decreased, their maxima moved at 394 and 450 nm and a new weak broadened absorption at 525 nm appeared (Figure 9A). These results suggest that the increase of the pH favored the photo-activation of **5**-cis. A further increase in the TEA concentration (0.5 mM) caused weak additional changes in the UV spectrum of **5** after irradiation (Figure 9B). The acquisition of time dependent UV spectra confirmed the reversibility of the conversion (Supplementary Figure S12).

Based on the above described results we explored the behavior of both the 5′-DMAzo-TBA and 5′-DMAzo-T30695 in a 5KEW blend containing 0.1 mM of TEA (5KEW-TEA) before and after the LED irradiation by means of standard UV spectroscopy. In order to assess the effect of the presence of TEA in the blend on the 5′-DMAzo-TBA and 5′-DMAzo-T30695 Qs, preliminary CD and fluorescence experiments were performed (Supplementary Figures S13 and S14). No significant changes were observed when comparing the results obtained with those described above for 5′-DMAzo-TBA and 5′-DMAzo-T30695 dissolved in 5KEW (Figure 6 and Supplementary Figure S5), thus confirming that the ability of the two sequences to fold into Qs was not significantly affected by the addition of TEA.

**Figure 10:**
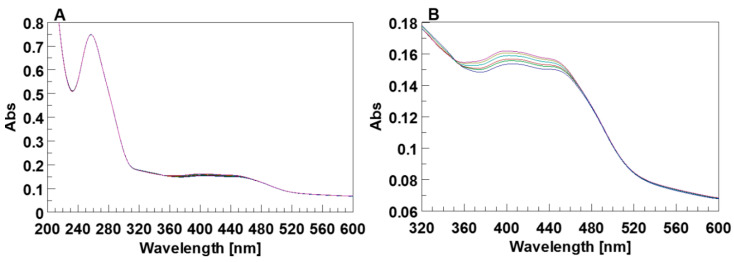
(**A**) Time-dependent UV spectrum of the 5′-DMAzo-TBA in the 5KEW-TEA blend; after irradiation at 436 nm. (**B**) The enlargement of the UV-vis region between 320–600 nm. The band intensities around 440 nm attributable to the absorption of the conjugated 5′DMAB slightly decrease after irradiation (blue line), and consecutively increases over time to reach the values observed before irradiation (violet line) in dark. [ON] = 3.5 × 10^−6^ M.

The UV spectra of 5′-DMAzo-TBA acquired before and after irradiation showed only weak reversible changes in the absorption bands at 405 and 454 nm, while the UV band around 260 nm was almost unchanged (Figure 10). Similar UV spectra acquired with the 5′-DMAzo-T30695 did not show any change after irradiation (Supplementary Figure S15). Therefore, the chemical environment around the DMAzo moiety (Figure 1) at the 5′-end of the two ONs could affect the acid–base equilibria of the dye in a manner in which neither the trans-cis conversion nor the effect of this conversion on the Qs were detectable with standard UV analyses, also using alkaline pH.

## 3. Discussion

In this study, two new TBA and T30695 variants have been synthesized through the conjugation at the 5′-end of each sequence of a DMAzo moiety. Our UV and CD studies showed that the new synthesized 5′-DMAzo-T30695, similarly to T30695, is able to fold into parallel Q structures. CD melting experiments conducted using both 10 K buffer and a 5KEW blend showed that the thermal stability of 5′-DMAzo-T30695-Q is higher than that of T30695-Q, thus suggesting that the DMAzo moiety has a favorable interaction with the Q structure. However, no further dichroic bands, besides those attributable to the T30695-Q, appeared in the spectra. Thus, neither the chirality of the Q, nor the chiral carbon in the glycerol linker on the DMAzo moiety caused a dichroic effect on the DMAzo absorption (350–500 nm).

Fluorescence experiments performed with an excitation at 255 nm showed that the conjugation partially suppressed the T30605-Q emission. It could speculate that this loss of fluorescence intensity might be due to quenching events involving the conjugated DMAzo. However, native PAGE experiments suggested that the presence of the DMAzo moiety at the 5’-end decreases the ability of T30695-Q to dimerize through 5′-5′ end-to-end stacking. Thus, similarly to the results obtained by adding a T residue at the 5′-end of T30695-Q [24,29,49], the partial suppression of the 5′-DMAzo-T30695-Q emitting properties with respect to those of T30695-Q could be attributable to the partial loss of the excimers formed through 5′-5′-end-to-end interactions between the Q monomers in the solution [49]. Significantly, in the 10 K buffer, irradiation at 450 nm of the 5′-DMAzo-T30695-Q caused an intense broad band emission around 550 nm. It is noteworthy that the 5’-DMAzo-T30695-Q emitted seven-fold more intensively than **5** dissolved in the same buffer. However, no fluorescence resonance energy transfer (FRET) phenomenon occurred when irradiating the sample at 255 nm (data not shown). Thus, the fluorescence features of the 5′-DMAzo-T30695-Q after irradiation at 450 nm should be connected to the influence of the chemical environment on the emitting properties of the conjugating dye.

The excitation at 255 nm of 5′-DMAzo-T30695 and T30695 annealed in the 5KEW blend caused an emission band around 310 nm in the fluorescence spectra. This emitting band was always observed for all the explored ONs and the increase in the temperature up to 95 °C caused only a partial suppression of its intensity, also in the case of the TBA, where the Q is certainly destroyed at 95 °C. On this basis, we hypothesized that the unstructured ONs in 5KEW may substantially contribute to this fluorescent band. 

In addition, only T30695-Q showed also two broad emission bands around 360 and 390 nm, which were very similar to those produced by the same Qs in the 10 K buffer. CD melting experiments showed that increase of the temperature from 25 to 95 °C of the T30695-Q solution caused only a partial loss (about 30%) in the intensity of the dichroic band at 263 nm. Furthermore, the acquired CD spectra in the temperature range 20–70 °C showed an isoelliptic point around 273 nm, thus suggesting that a transition between the two Q species, namely bimolecular→monomolecular Qs was occurring in the solution [24,29]. The fact that the intensity of the positive dichroic band at 263 nm still appeared at 90 °C suggested that T30695-Q monomers were still in the solution while the corresponding dimers were destroyed by heating up to 70 °C. Meanwhile, with an increase in the temperature from 25 to 65 °C the fluorescence intensity of the broad bands at 360 and 390 nm decreased. Based on these results, it can be inferred that the T30695-Q dimers could be responsible for these emission bands.

Parallel CD and fluorescence spectral measurements performed on the 5’-DMAzo-T30695-Q showed that the increase in the temperature from 20 to 90 °C did not significantly affect the CD band at 263 nm whereas at 25 °C only weak emissions at 360 and 390 nm after excitation at 255 nm were observed. These results suggested that similarly to the 10 K buffer, also in the 5KEW blend the presence of DMAzo at the 5′-end of the T30695-Q could affect the ability of T30695 to form Q dimers. Furthermore, with an irradiation of the 5′-DMAzo-T30695-Q in 5KEW at 450 nm and an increase in the temperature from 25 to 75 °C the fluorescence spectra showed a significant change in the emission bands, and an isofluorescent point at about 525 nm appeared. Considering that this phenomenon is generally due to the transition between two differently emitting states of a given molecule [61], the increase in the temperature could cause the loss of such interactions between the dye and the T30695-Q.

More complex events govern the folding 5′-DMAzo-TBA into Qs. Indeed, the conjugation of dye at the 5′-end of the TBA caused a significant change in the ability of the sequence to form a monomolecular Q, as evidenced by electrophoresis experiments on non-denaturing gel. The 5′-DMAzo-TBA annealed in the 100 K buffer or 5KEW blend always showed a high number of bands having low electrophoretic mobilities, in addition to a weak band having the same electrophoretic mobility as the TBA. These data indicated that the presence of the dye induced the shift of the TBA folding from a monomolecular- to multimeric- Q. In line with these results, previously reported studies have demonstrated the sensitivity of the TBA-Q typology to such specific modifications [62]. In order to obtain additional information about the structural features of the 5′-DMAzo-TBA-Qs fluorescence experiments were also performed irradiating at 255 nm the 5′-DMAzo-TBA or TBA in both the 100 K buffer and 5KEW. However, the resulting spectra did not exhibit any significant differences. The overall results of the CD and fluorescence experiments suggested that 5′-DMAzo-TBA-Q aggregates could preserve certain TBA-Q features, such as the antiparallel orientation of the strands. 

As had occurred in relation to the 5′-DMAzo-T30695, the excitation at 450 nm of the 5′-DMAzo-TBA gave rise to different fluorescence features depending on the solvent conditions used. In the 100 K buffer a broad fluorescent band emission centered at 545 nm appeared, whereas in the 5KEW blend the maximum of the main band was around 497 nm with a shoulder at 530 nm. In both cases the fluorescence intensity of the 5′-DMAzo-TBA was significantly higher than that of the free dye. Furthermore, the substantial differences observed when comparing the fluorescence data acquired on the 5′-DMAzo-TBA with those obtained for 5′-DMAzo-T30695 indicated that the conjugation to the Qs always positively impacted on the fluorescence intensity acquired after excitation at 450 nm and that the emitting profiles were related to the Q typology carrying the dye at the 5′-end as well as to the solvent system used.

Finally, in order to explore the trans-cis conversion of the dye incorporated at the 5′-end of the TBA and T30695, a preliminary investigation into the behavior of the free dye under irradiation by LED 436 nm was performed by means of UV spectroscopy. As a general rule, the trans-cis conversion of diazobenzene and its analogues can be quickly proved through the acquisition of UV spectra before and after irradiation at specific wavelengths [50]. The acquisition of time-dependent UV spectra provides for the reversibility of the trans to cis diazobenzene conversions and for the kinetic parameters of the reaction. Different phenomena can affect the kinetics of the trans-cis conversion and of the reverse reaction, such as the polarity of the solvents, the type and position of the functional groups on the aromatic rings, and the presence of acid-base equilibria. Both theoretical and experimental studies suggest that the protonation of the diazo-group of the free DMAzo analogues occurs also at neutral pH in protic solvents, as it proceeds through tautomeric equilibria involving the dimethyl-amino group on one phenyl ring [36,51,52,53,54,55]. Due to the reduction of the double bond degree in the protonated diazo group, the thermal decay of the cis DMAzo analogues occurs from ns to s, depending on the existence of further functional group on the benzene rings and on the solvent used. However, in line with previously reported data [36,51,52,53,54,55], we evidenced that TEA is effective in inducing an increase in the half-life of the free cis-dye in 5KEW, allowing us to obtain significant and reversible changes in the standard UV spectra acquired before and after LED irradiation. 

Despite this, LED irradiation did not affect the UV spectrum of 5′-DMAzo-T30695, and only small reversible changes in that of the 5′-DMAzo-TBA were noticeable. As a consequence of the presence of a sufficiently extended aromatic core in the dye, we speculated that the thermodynamically stable trans geometry of the dye was further stabilized by stacking interactions with the neighboring G-quartet, thus affecting the energetic gap between trans-cis isomers. However, differently from typical Q ligands having large aromatic ring systems, such as BRACO-19 [63] or pyridostatin [64], the conjugated dye was not able to stack on all four guanine bases of the G-tetrad, thus allowing limited stacking interactions. As a consequence, in alkaline medium, the trans-to-cis conversion should be detectable, as had occurred for double helices incorporating DMAzo analogues [17,56,57]. In addition, the dye was not functionalized with specific substituents able to support the stacking on the G-quartet by additional interactions [65,66,67,68,69,70]. Based on these observations, we hypothesized that such interactions between the dye and the Qs can stabilize a specific tautomer of the protonated dye, thus accelerating or hampering the trans-cis conversion also using alkaline pH [51,52,53,54,55]. Further detailed studies are currently in progress to corroborate our hypothesis. The characterization of the structural behavior of the DMAzo conjugating Qs will be useful in order to obtain new insights concerning the conditions that govern the fluorescence enhancement of the DMAzo conjugating Qs.

## 4. Materials and Methods

### 4.1. General

Commercial reagents, all organic solvents and water (HPLC grade) were purchased from Merck (Kenilworth, NJ, USA). Silica gel chromatography was performed using Merck silica gel 60 (0.063–0.200 mm). TLCs were run on Merck silica gel 60 F254 plates (Kenilworth, NJ, USA) and the spots were visualized by means of an UV lamp (Vilber Lourmat VL-4LC, 365 and 254 nm). CD experiments were performed on a JASCO 715 spectropolarimeter equipped with a PTC-348 temperature controller. ON syntheses were performed on a PerSeptive Biosystem Expedite DNA synthesizer. HPLC purifications and analyses were carried out using a JASCO PU-2089 Plus HPLC pump equipped with a JASCO BS-997-01 UV detector. UV experiments were performed on a JASCO V-530 spectrophotometer, equipped with a PTC-348 temperature controller. ^1^H (500 MHz and 700 MHz) and ^13^C (125 MHz). NMR spectra were recorded on an Agilent INOVA spectrometer (Agilent Technology, Cernusco sul Naviglio, Italy); chemical shifts were referenced to the residual solvent signal (CD_3_OD: *δ*_H_ = 3.31, *δ*_C_ = 49.0 ppm; DMSO-*d*_6_: *δ*_H_ = 2.50, *δ*_C_ = 39.5 ppm). For an accurate measurement of the coupling constants, the one-dimensional ^1^H NMR spectra were transformed at 64 K points (digital resolution: 0.09 Hz). HRMS (ESI positive mode) was performed with a Thermo LTQ Orbitrap XL mass spectrometer (Thermo-Fisher, San Josè, CA, USA). The spectra were recorded by infusion into the ESI source using MeOH as solvent.

### 4.2. Chemistry

Compounds **2** and **3** were synthesized and characterized according to previously reported procedures [35]. 

*(R, E)-3-(4-((4-(dimethylamino)phenyl)diazenyl)phenoxy)propane-1,2-diol (****5****).* A mixture of **3** (0.400 g, 2.2 mmol), 205 µL fluoroboric acid (water solution, 48%, 3.3 mmol) and 4 mL of EtOH was cooled to −15 °C under argon and then 224 µL of isoamyl nitrite (0.258 g, 2.2 mmol) were added. After 12h under stirring, the produced diazonium salt was first precipitated by diluting the mixture with n-hexane, and then further washed with n-hexane (three times). The solid residue was dried under vacuum to give **4**, which was used in the next step without further treatment. Compound **4** (0.360 g, 1.8 mmol) was dissolved in 10 mL of acetic acid (14.0 g, 232 mmol), under magnetic stirring. Sodium acetate (4.0 g, 511 mmol) and dimethylaminoaniline were consecutively added to the solution. After 15 min at r.t., the formation of a gel-like mixture was observed. The reaction time was further prolonged up to 1 h, under slow stirring. The final mixture was diluted with water and extracted with DCM (4–5 times). The collected organic layers were dried under vacuum and the residue re-dissolved in MeOH. The obtained solution was embedded on silica and then rotary evaporated to dryness. The resulting powder was applied on a DCM: propan-2-ol 90:10 (v/v) packed silica gel column and then chromatographed using isocratic conditions. The collected fractions, dried under vacuum (TLC 90:10 DCM:MeOH, v/v, R_f_ = 0.6), were further purified on HPLC RP-18 column (H_2_O:CH_3_CN, 80:20 v/v, t_R_ = 12 min), to get **5** (69% yields).

^1^H NMR (500 MHz, CD_3_OD) *δ*: 3.06 (s, 6H, N(CH_3_)_2_), 3.67 (m, 2H, CH_2_OH), 4.00 (m, 1H, CHOH), 4.04 (dd, *J* = 4.2, 9.6 Hz, 1H, OCH_2_), 4.14 (dd, *J* = 6.0, 9.6 Hz, 1H, OCH_2_), 6.82 (d, *J* = 9.1 Hz, 2H, C^3,5^_Ar_H), 7.06 (d, *J* = 8.9 Hz, 2H, C^2,6^_Ar_H), 7.77 (d, *J* = 9.1 Hz, 2H, C^2,6^_Ar_H), 7.77 (d, *J* = 8.9 Hz, 2H, C^3,5^_Ar_H). ^13^C NMR (125 MHz, CD_3_OD) *δ*: 39.3 (N(CH_3_)_2_), 62.9 (CH_2_OH), 70.6 (CHOH), 69.4 (OCH_2_), 111.5 (C^3,5^_Ar_), 114.6 (C^2,6^_Ar_), 123.8 (C^3,5^_Ar_), 124.3 (C^2,6^_Ar_), 143.7 (C^1^_Ar_), 147.7 (C^4^_Ar_), 152.5 (C^4^_Ar_), 160.7 (C^1^_Ar_). [α]^25^= -21.5 [c= 21,6 mg/mL; EtOH]

Synthesis of phosphoramidite building blocks (**7**): Compound **5**, (0.400 g, 1.3 mmol), 4,4-dimethoxytrityl chloride (0.430 g, 1.3 mmol) and 4-dimethylaminopyridine (0.0076 g, 0.062 mmol) were dissolved in dry pyridine (6.35 mL). The resulting solution was stirred at room temperature (r.t.) under argon for 3 h. The solution was concentrated under reduced pressure and the residue purified by column chromatography on silica gel (eluted with 50:50:0.1 N hexane/ethyl acetate /Et_3_N) to give mono-dimethoxytritylated **6** as a clear orange solid (yields 44%). 

Compound **6** (0.350 g, 0.57 mmol), *β*-cyanoethyl-*N*,*N*’-diisopropylchlorophosphoramidite (0.190 mL, 0.85 mmol) and DIPEA (0.296 mL, 1.7 mmol) were dissolved in dry DCM (3 mL), 1.0 h. After 60 min, the reaction was diluted with ethyl acetate (15 mL), and finally washed with sodium carbonate solution (10%, 15 mL) and brine (15 mL). The organic layer was dried on magnesium sulphate and concentrated in vacuo. The residue was purified by silica gel chromatography eluted with hexane/ethyl acetate and triethylamine (80:10:10). The fractions containing the product were collected and concentrated under vacuum, yielding **7** as an orange foam (85% yield). ^1^H, ^13^C, ^31^P NMR and HRMS of the final product are reported in the supplementary material (Supplementary Figures S16–S19).

Synthesis of conjugated aptamers: The TBA and T30695 were synthesized using standard solid phase DNA chemistry on a controlled pore glass (CPG) support 3′-bounded to the first nucleotide of the sequence, following the β-cyanoethyl phosphoramidite method. The corresponding conjugated sequences were obtained by adding a manual coupling stage with the phosphoramidite building block **7** at the end of the synthesis of the TBA and T30695 sequences. The coupling time of the final two coupling steps was extended from 2 to 20 min. The resulting DMAzo 5’-end conjugated TBA and T30695 sequences were finally detached from the solid support by treatment with 20% aqueous ammonia at rt for 18 h. The combined filtrates and washings were concentrated under reduced pressure, dissolved in H_2_O, and purified by HPLC using an anionic exchange column (Nucleogel SAX, Macherey-Nagel) eluted with a linear gradient (from 0 to 80% B in 30 min) of phosphate buffer at pH 7.4 (A:20 mM NaH_2_PO_4_ aqueous solution containing 20% CH_3_CN; B: 1.0 M NaCl, 20 mM NaH_2_PO_4_ aqueous solution containing 20% CH_3_CN. Elution times: 19.0 min T30695; 22.0 min 5′-DMazo-T30695; 16.5 min TBA; 18.0 min 5′-DMazoTBA). The oligomers were successively desalted by molecular exclusion chromatography on Biogel P-2 Fine. The purity (95%) was checked by HPLC on an RP-18 column (Merk Purosphere-Star) eluted with a linear gradient of CH_3_CN (from 0 to 30% B in 40 min) in triethylammonium acetate (TEAA) buffers (Elution times: 9.0 min T30695; 11.0 min 5′-DMazo-T30695; 6.0 min TBA; 9.0 min 5′-DMazoTBA). The concentrations of the samples were determined by measuring the absorbance at 260 nm at 90 °C and using the open access program available on http://basic.northwestern.edu/biotools/OligoCalc.html. 

### 4.3. UV Experiments

The UV spectra of the free DMAzo, T30695, 5′-DMAzo-T30695, TBA and 5′-DMAzo-TBA, in 10K (5 mM potassium phosphate, 5 mM KCl, pH = 7.4) or 100 K (10 mM potassium phosphate,90 mM KCl, pH = 7.4) buffers or in H_2_O/EtOH (1:1,v:v) containing 5.0 mM KCl (5KEW) were acquired at ON concentrations of 20 or 2.0 µM, respectively, using cuvette o.l. 0.5 cm Vol. 1400 µL or 0.1 cm Vol. 400 µL. All samples were annealed by heating the solution at 90 °C for 10 min and then gently cooling the solutions at room temperature. Finally, the UV spectra were also acquired by adding to the samples in 5KEW blend triethylamine (TEA) at a final concentration of 100 µM. LED (Roithner-Laser Tech) at 436 nm (three LED on electronic place, Power 12 mV, I = 12 mA) was used to irradiate the samples put in the UV cuvette, o.l. 0.5 cm, Vol = 1400 µL). The time-dependent UV spectra of the free DMAzo, 5′-DMAzo-T30695 and 5′-DMAzo-TBA in the 5KEW-TEA blend were obtained by acquiring the UV spectrum of the irradiated samples immediately after the LED irradiation and, successively, at a time interval of 2 min.

### 4.4. CD and CD Melting Experiments

The CD spectra of T30695, 5′-DMAzo-T30695, TBA and 5′-DMAzo-TBA were acquired at ON concentrations of 20 or 2.0 µM on samples obtained as above described for the UV experiments. Cuvette o.l. 0.5 cm Vol. 1400 µL All the samples were annealed by heating at 90 °C for 10 min and then gently cooling the solutions at room temperature. The temperature-dependent CD spectra of the ONs were acquired in the range 20–60 °C for TBA and 5′-DMAzo-TBA (T = 20, 30, 40, 50, 55, 60 °C) and in the range 20–90 °C for T30695 and 5′-DMAzo-T30695. (T = 20, 30, 40, 50, 60, 70, 80, 90 °C). Each sample was equilibrated for 30 min at the specific temperature inside the thermal cell of the instrument before starting the scan; at each temperature, the spectrum was the result of the average of three acquisitions. CD melting experiments were performed monitoring the change of the intensity of the band at 263 (T30695 and 5′-DMAzo-T30695) or 295 nm (TBA and 5′-DMAzo-TBA) increasing the temperature from 10 to 100 °C or from 10 to 90 °C. For each sample, the ON concentration was 20 µM. The temperature scan speed was 0.1 °C/min. 

### 4.5. Fluorescence Spectroscopy

The steady-state fluorescence measurements were performed on a PC1 Fluorometer (ISS, Champaign, IL, USA) equipped with a 2-cell temperature-controlled sample holder. The ON samples and **5** (Scheme 1) were dissolved, in 10 K or 100 K buffers, 5 KEW or 5 KEW-TEA blends at the same concentration as for the UV experiments (for details see the corresponding section) and diluted at the final values of 0.1 OD at the maximum value of the excitation wavelength. The temperature of the samples was measured directly in the cuvette with an accuracy of 0.2 °C. The normalized data were obtained by dividing the relative fluorescence intensity at each wavelength by the relative fluorescence intensity at the peak wavelength. 

Intrinsic fluorescence emission of conjugated aptamers: In order to study the intrinsic fluorescence emission of the synthesized molecules, steady-state fluorescence measurements were performed. The excitation wavelength was stetted at 255 nm and the emission spectra were recorded in the range 300–460 nm. A total volume of 400 μL was used and the excitation and emission slit widths were set at 1.0 and 0.5 nm respectively.

Un-conjugated and conjugated DMAzo fluorescence intensity characterization: The fluorescence characterization of un-conjugated and conjugated DMAzo compound was performed. For this purpose, steady state measurements were executed on the samples prepared in the 10 K, 100 K or 5KEW blends. The excitation wavelength was stetted at 450 nm and the emission spectra were recorded in the range 480–650 nm.

*TEA effect on the fluorescence emission*: The TEA effect on the 5′-DMAzo-T30695, 5′-DMAzo-TBA and DMAzo fluorescence emission was evaluated. The samples diluted in the 5KEW-TEA blend were excited at 255 and 450 nm respectively. The spectra were recorded at 25 °C.

*Fluorescence emission melting experiments*: Fluorescence emission melting experiments were performed to monitor the change in the fluorescence intensity when the T30695, TBA, 5′-DMAzo-T30695, 5′-DMAzo-TBA and DMAzo were excited at 255 and 450 nm respectively. The range of temperatures investigated was from 25 to 95 °C in the 5KEW or the 5KEW-TEA blends.

Before each measurement the sample was incubated for 10 min at the single temperature value and the spectra were acquired three times.

### 4.6. Electrophoresis Gel Shift Assay

The oligonucleotides were annealed in the 10 K (T30695, 5′-DMAzo-T30695, T30695-TT) or 100 K (TBA and 5′-DMAzo-TBA) buffer or the 5KEW blend. The samples, with a total strand concentration of 0.1 O.D. × μL (100 μL), were heated to 90 °C, gently cooled at r.t., then incubated at 20 °C for two days. Subsequently, 5.0 µL of each oligonucleotide were mixed with 5.0 µL of glycerol and the resulting solutions were loaded on 12% non-denaturing polyacrylamide gels and run in 1× TBE buffer containing 30 or 5.0 mM of KCl, at 5 °C for 5 h at 120 V. The gel was visualized by means of a UV lamp.

## Supplementary Materials

Supplementary materials can be found at https://www.mdpi.com/1422-0067/21/19/7103/s1.

## Abbreviations

Q	Guanine Quadruplex
GRO	G-quadruplex rich oligonucleotide
CPG	Controlled pore glass
ON	oligonucleotide
DMAzo	(*R*) 4-(dimethylamino)azobenzene glycerol derivative
DMT	4,4′-dimethoxytrityl protecting group
TBA	thrombin binding aptamer
TEA	triethylamine
Py	pyridine
10 K	10 mM of potassium phosphate at pH = 7.4
100 K	10 mM of phosphate containing 90 mM of KCl at pH = 7.4
5KEW	EtOH: H2O 1:1 v/v containing 5 mM of KOH
5KEW-TEA	5KEW blend containing 0.1 mM of TEA
TEAA	triethylammonium acetate
DIPEA	*N*,*N*-Diisopropylethylamine
DCM	dichloromethane
