# Supplementary material for: Spectroscopic Properties of Two 5′-(4-Dimethylamino)Azobenzene Conjugated G-Quadruplex Forming Oligonucleotides
Source: Int J Mol Sci. 2020 Sep 26;21(19):7103. doi: 10.3390/ijms21197103 (PMC7582650; doi:10.3390/ijms21197103)
Supplement: Supplementary file 1 [file ijms-21-07103-s001.pdf]

Supplementary Information

Spectroscopic Properties of Two 5'-(4-dimethylamino)azobenzene Conjugated G-Quadruplex Forming Oligonucleotides

Concetta Imperatore [1], Antonio Varriale [2], Elisa Rivieccio [1], Angela Pennacchio [2], Maria Staiano [2], Sabato D'Auria [2], Marcello Casertano [1], Carlo Altucci [3], Mohammadhassan Valadan [3], Manjot Singh [3], Marialuisa Menna [1], Michela Varra [1,*]

[1] Department of Pharmacy, School of Medicine, University of Naples "Federico II", Via D. Montesano 49, 80131, Naples, Italy; cimperat@unina.it (C.I.); elisrivi90@gmail.com (E.R.); marcello.casertano@unina.it (M.C.); mlmenna@unina.it (M.M.);

[2] Institute of Food Sciences, National Research Council of Italy, via Roma 64, 83100, Avellino, Italy; antonio.varriale@isa.cnr.it (A.V.); angela.pennacchio@isa.cnr.it (A.P.); maria.staiano@isa.cnr.it (M.S.); sabato.dauria@cnr.it (S.D.);

[3] Department of Physics "Ettore Pancini", University of Naples Federico II, Via Cinthia, 21 - Building 6, 80126, Naples, Italy; mohammadhassan.valadan@unina.it (M.V.); manjot.singh@unina.it (M.S.); altucci@na.infn.it (C.A.);

*Correspondence: varra@unina.it (M.V.); Tel.: +39-081-678540

Figure S1. UV spectra of TBA and 5'-DMAzo-TBA in 100K buffer (pH=7.4), and of T30695 and 5'-DMAzo-T30695 in 10K buffer (pH=7.4).

Figure S2. UV spectra of TBA, 5'-DMAzo-TBA, T30695 and 5'-DMAzo-T30695 in 5KEW.

Figure S3. CD spectra of TBA, 5'-DMAzo-TBA , T30695 and 5'-DMAzo-T30695 in 5KEW.

Figure S4. CD spectra at 20 and 90 °C of T30695 and 5'-DMAzo-T30695 in 5KEW blend.

Figure S5. Temperature-dependent CD spectra in 5KEW blend of TBA, 5'-DMAzo-TBA, T30695 and 5'-DMAzo-T30695.

Figure S6. Fluorescence spectra of 5'-DMAzo-TBA (100K), 5'-DMAzo-T30695(10K) and DMAzo (10K) excited at 450 nm.

Figure S7. Fluorescence spectra at 25 °C of T30695 and 5'-DMAzo-T30695 and TBA and 5'-DMAzo-TBA excited at 255 nm.

Figure S8. Fluorescence spectra of free DMAzo, 5'-DMAzo-TBA at different temperatures (25-95°C), excited at 450 nm and acquired in the region 480-650 nm.

Figure S9. Fluorescence spectra of 5'-DMAzo-T30695 in 5KEW blend at 25-55 °C and 65-95°C excited at 450 nm.

Figure S10. EMSA on non-denaturating gel of unmodified and conjugated ONs annealed in 5KEW blend.

Figure S11. UV spectra of DMAzo in 5KEW before and after excitation with LED at 436 nm and after addition of TEA.

Figure S12. Time-dependent UV spectra of free DMAzo in 5KEW-TEA blend after irradiation with LED at 436 nm.

Figure S13. CD and CD-melting of 5'-DMAzo-TBA and 5'-DMAzo-T30695 in 5KEW-TEA blend.

Figure S14. Comparison of fluorescence spectra of 5'-DMAzo-TBA and 5'-DMAzo-T30695 in 5KEW blend with those performed in 5KEW-TEA blend excited at 255 nm.

Figure S15. Time-dependent UV spectra of 5'-DMAzo-T30695 in 5KEW-TEA blend before and after the irradiation.

Figure S16. ^{1}H NMR Spectrum DMAzo phosphoramidite building block.

Figure S17. ^{31}P NMR Spectrum DMAzo phosphoramidite building block.

Figure S18. ^{13}C NMR Spectrum DMAzo phosphoramidite building block.

Figure S19. HRMS DMAzo phosphoramidite building block.

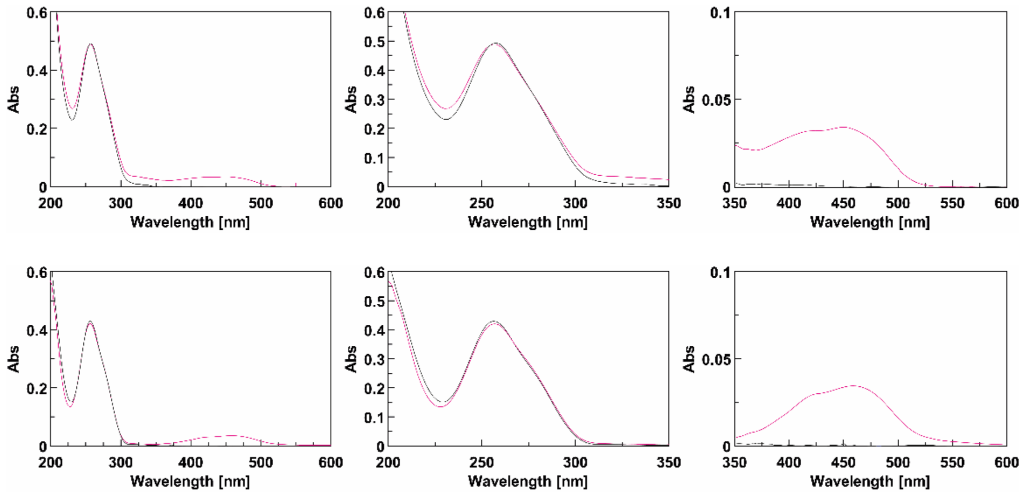

Figure S1. **A**. UV spectra of TBA (black line) and 5’-DMAzo-TBA (fucshia line) in 100K buffer (pH=7.4) acquired in the range of 200-600 nm. **B.** UV spectra of T30695 (black line) and 5’-DMAzo-T30695 (fucshia line) in 10K buffer (pH=7.4), in the range of 200-600 nm. **A’, A’’** and **B’**, **B’’** The rescaling to the range of 200-400 nm and to the range 350-600 nm of A and B, respectively**.** The spectra were acquired at 10°C using [ON]= 20 μM, cuvette o.l. 0.1 cm, V=400 μL.

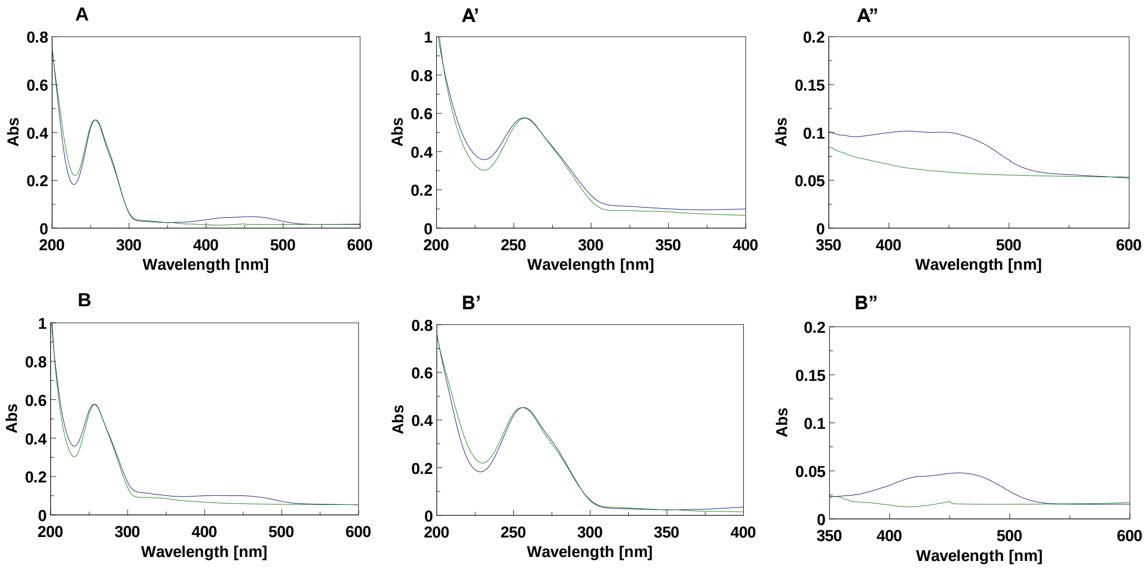

Figure S2. **A**. UV spectra of TBA (green line) and 5’-DMAzo-TBA (blue line) in 5KEW blend in the range of 200-600 nm. **B.** UV spectra of T30695 (green line) and 5’-DMAzo-T30695 (blue line) in 5KEW blend, in the range of 200-600 nm. **A’, A’’** and **B’**, **B’’** The rescaling to the range of 200-400 nm and to the range 350-600 nm of A and B, respectively. The spectra were acquired at 10°C using [ON]= 2.0 μM, cuvette o.l. 1.0 cm, V=1400 μL.

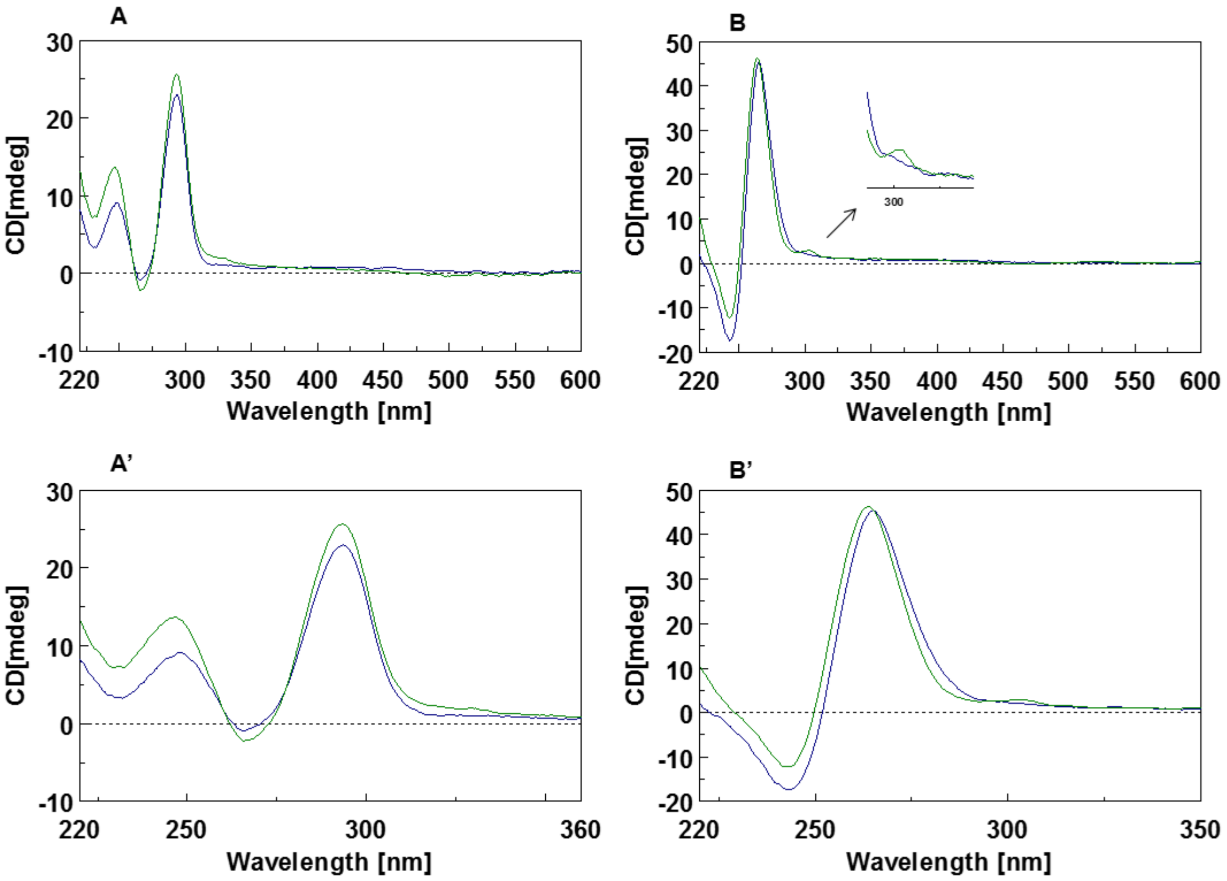

Figure S3. CD spectra of **A** TBA (green) and 5'-DMAzo-TBA (blue), and **B** of T30695 (green) and 5'-DMAzo-T30695 (blue), acquired in 5KEW. **A'** and **B'**. The rescaling to the range of 220-350 nm of A and B, respectively. The spectra were acquired at 10 °C using [ON]= 2.0 µM. Cuvette o.l. 1.0 cm, Vol=1400 µL.

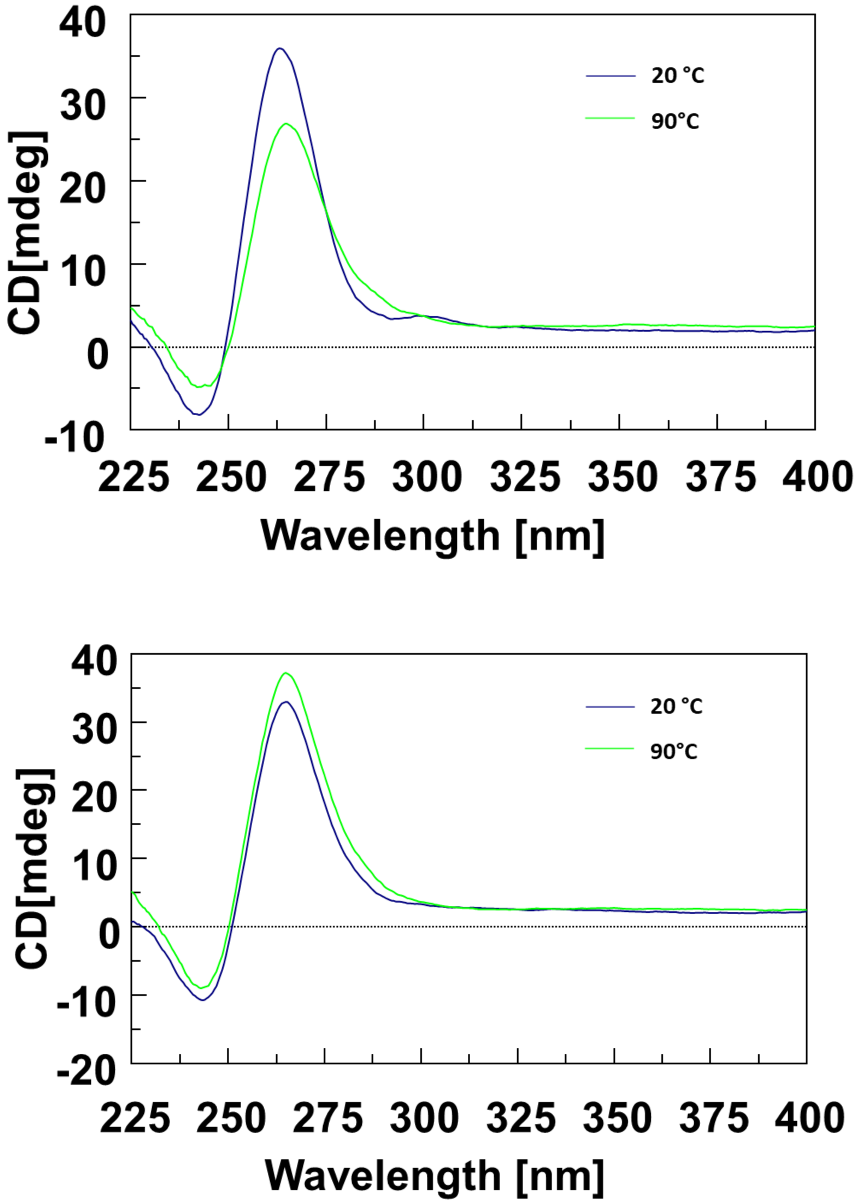

Figure S4. CD spectra at 20 and 90 °C for T30695 (**A**) and 5’-DMAzo-T30695 (**B**) respectively. In all cases ONs were used at final concentration of 2.0 µM in 5 KEW blend.

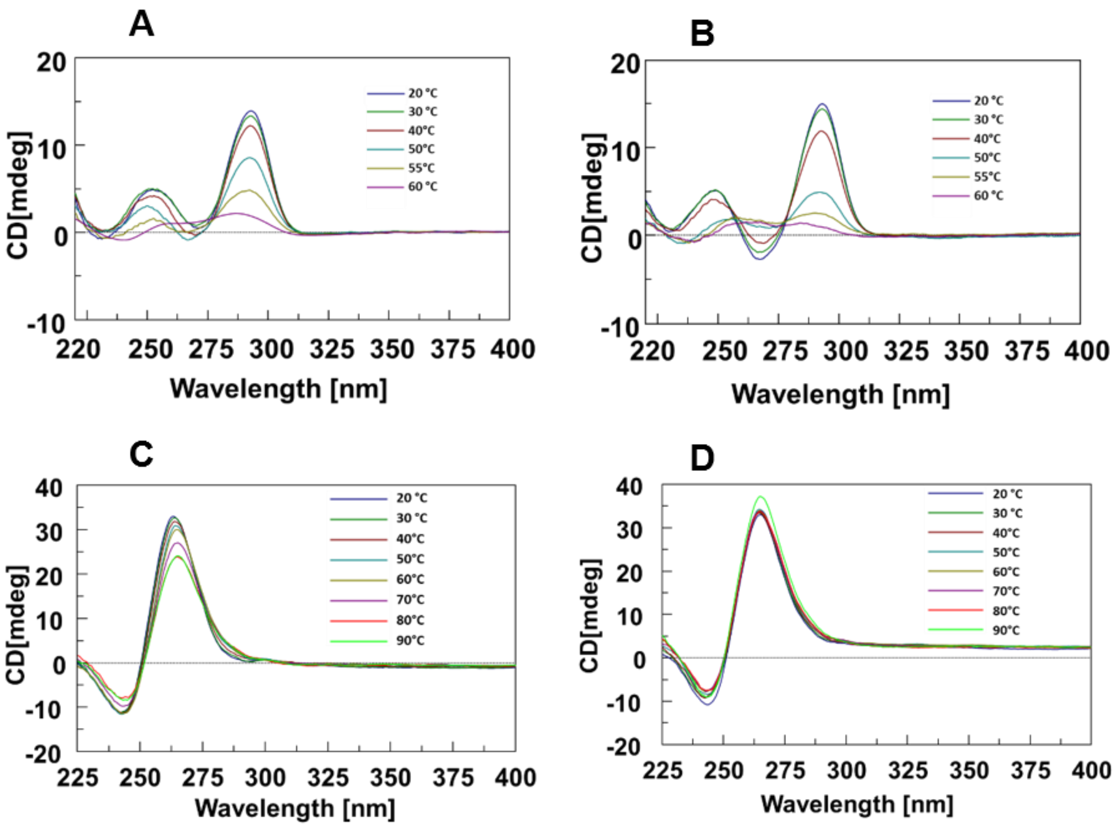

Figure S5. Temperature-dependent CD spectra in 5KEW blend of **A**. TBA; **B.** 5′-DMAzo-TBA; **C.** T30695; **D.** 5′-DMAzo-T30695. At each temperature, the CD profile was obtained subtracting the CD spectrum of 5KEW blend alone from that of the corresponding ON solution. The spectra were acquired at 10 °C using [ON]= 2.0 μM. Cuvette o.l. 1.0 cm, Vol=1400 μL.

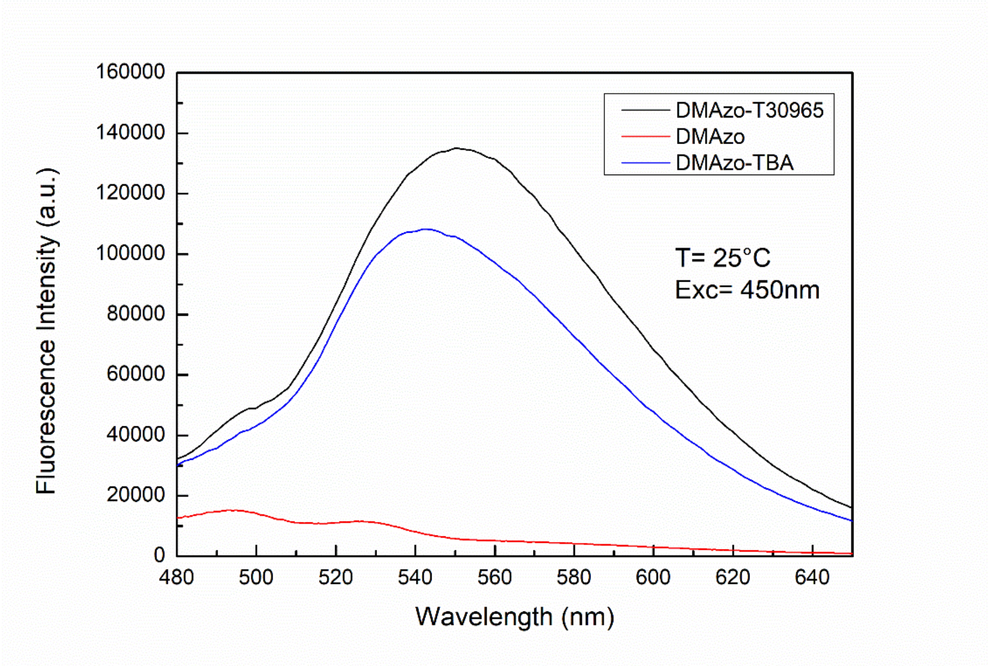

Figure S6. Fluorescence spectra of 5’-DMAzo-TBA (in 100K buffer), 5’-DMAzo-T30695(in 10K buffer) and DMAzo (in 10K buffer) excited at 450 nm and acquired in the range 480-650 nm. 100K buffer:10 mM of phosphate buffer containing 90 mM of KCl (pH=7.4). 10K buffer: 10 mM of potassium phosphate buffer (pH=7.4).

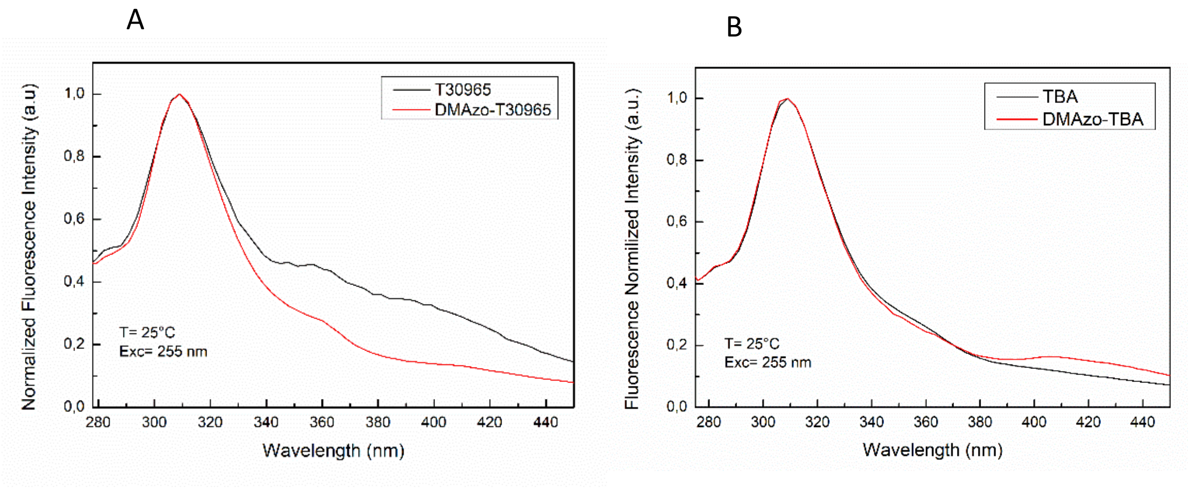

Figure S7. Fluorescence spectra at 25 °C of T30695 and 5’-DMAzo-T30695 (**A**) and TBA and 5’-DMAzo-TBA (**B**) excited at 255 nm acquired in the region 300-460 nm. In all cases 5KWE was used as a solvent.

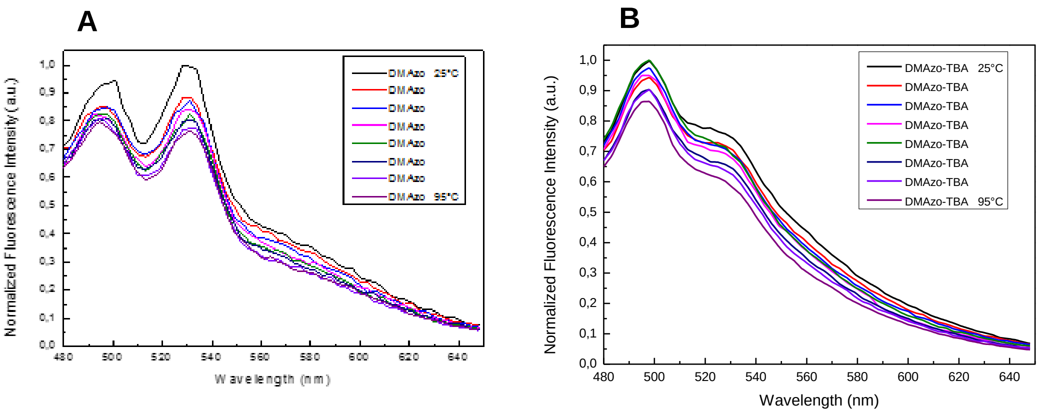

Figure S8. Fluorescence spectra of free DMAzo (A) and 5'-DMAzo-TBA (B), at different temperatures (25-95°C), excited at 450 nm and acquired in the region 480-650 nm. In all cases 5KWE was used as a solvent.

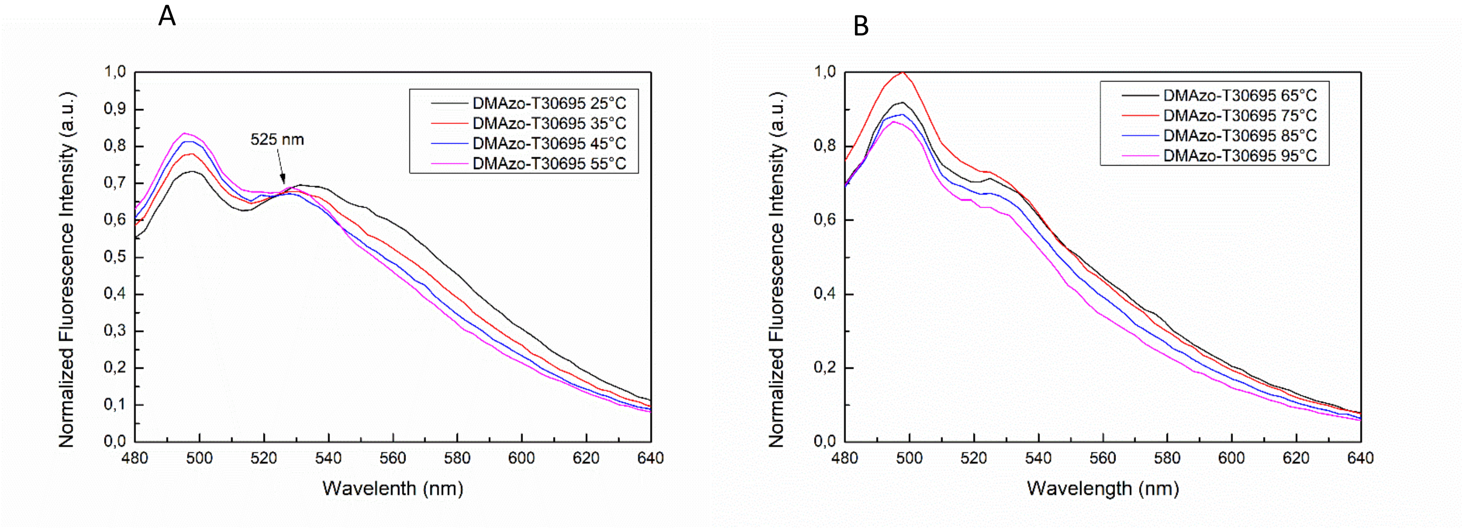

Figure S9. Fluorescence spectra of 5'-DMAzo-T30695 excited at 450 nm and acquired in the region 480-650 nm at 25-55 °C (**A**) and 65-95°C (**B**). In all cases 5KWE was used as a solvent.

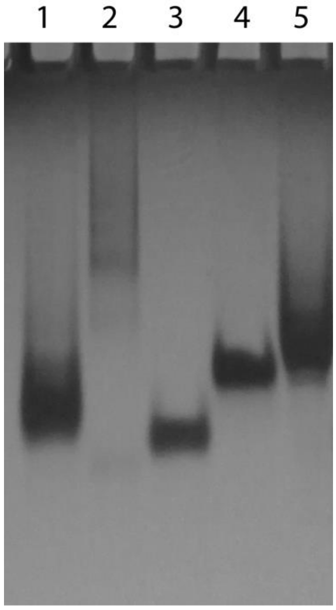

Figure S10. Electrophoresis mobility shift assay of conjugated and unmodified sequences run on non-denaturating acrylamide bis-acrylamide gel (16%) containing 30 mM KCl. All samples were annealed in 5KEW bland. Gel was run at 8-10 °C. Lane 1: 5'-TT-T30695; lane 2: 5'-DMAzo-TBA; lane 3: TBA; lane 4: 5'-DMAzo-T30695; lane 5: T30695.

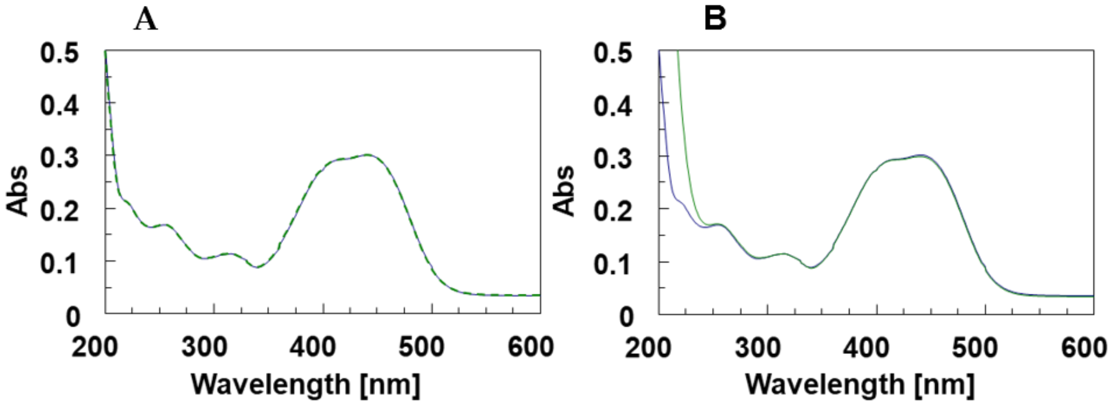

Figure S11. UV spectra of A. DMAzo in 5KEW before (dashed green line) and after (blue line) excitation with LED at 436 nm, and B. DMAzo in 5KEW before (blue line) and after (green line) addition of TEA at final concentration of 0.1 mM.

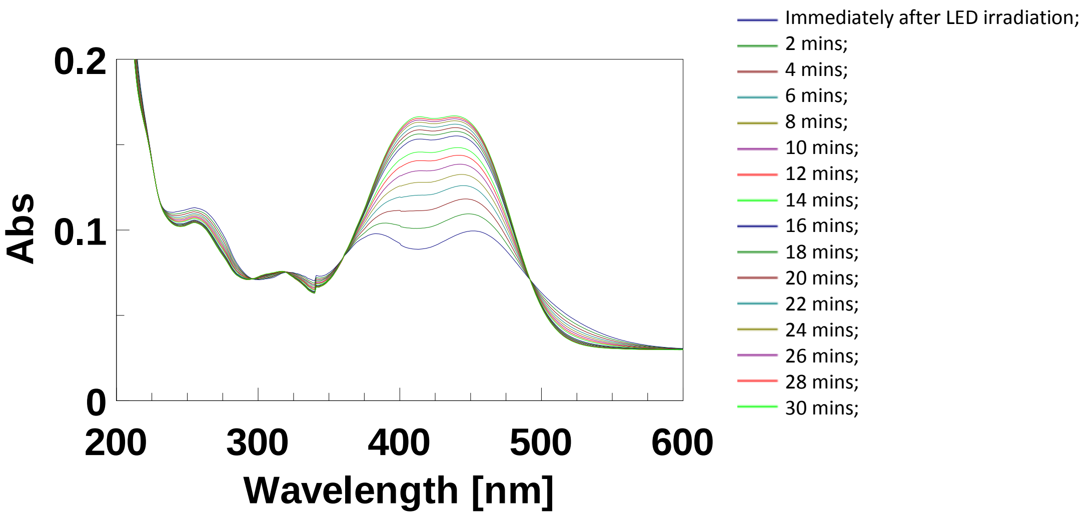

Figure S12. Time-dependent UV spectra of free DMAzo (**5**, scheme 1 in the manuscript) in 5KEW-TEA blend after irradiation with LED at 436 nm. The spectra were acquired every two minutes until the spectrum showed the same profile than that acquired in the dark.

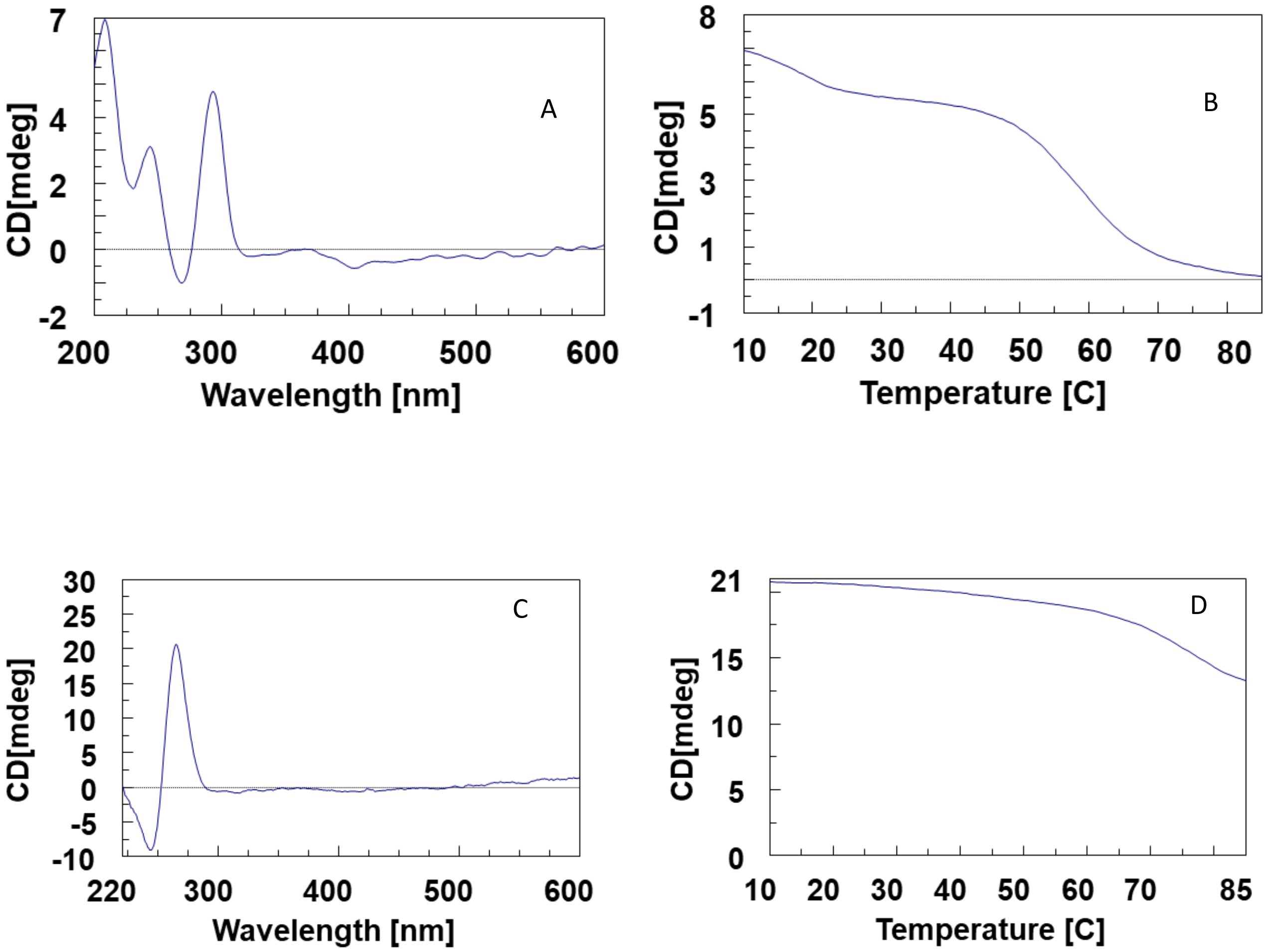

Figure S13. **A** and **B**. CD and CD-melting (acquired monitoring the amplitude of the CD band at 295 nm) of 5’-DMAzo-TBA in 5KEW-TEA blend. **C** and **D**. CD and CD-melting (acquired monitoring the amplitude of the CD band at 263 nm) of 5’-DMAzo-T30695 in 5KEW-TEA blend. 5’-DMAzo-T30695 Q appeared to be not completely unfolded in the range of explored temperatures.

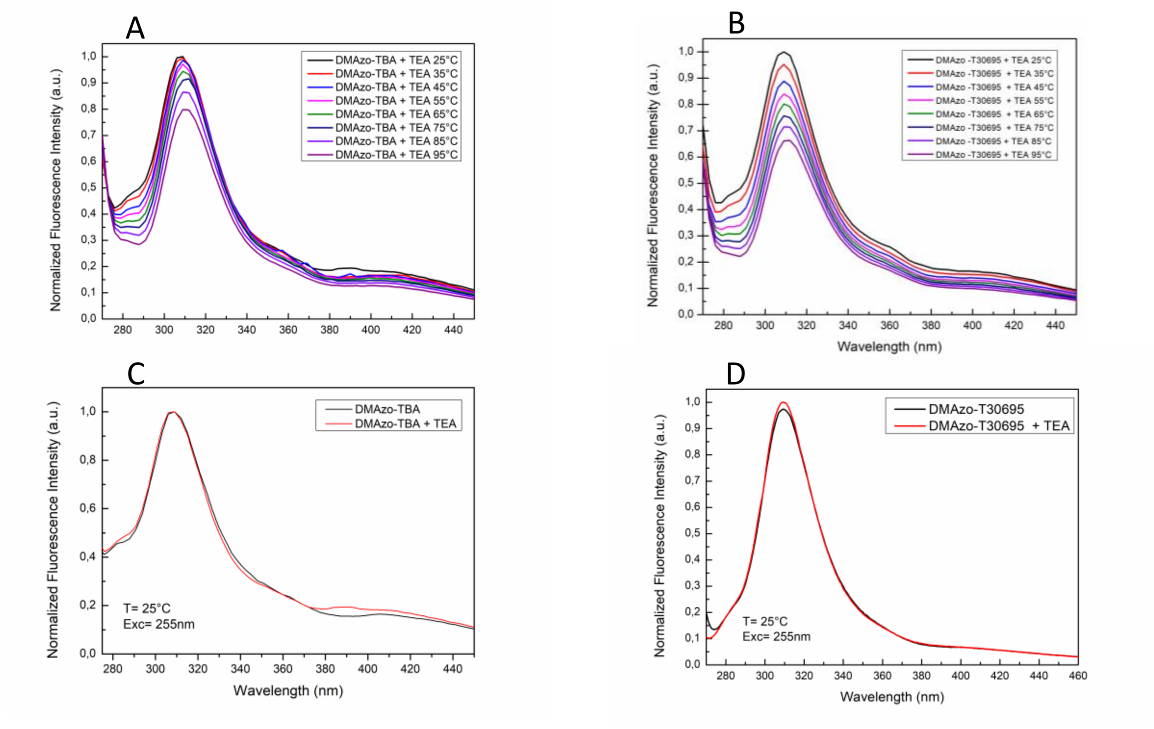

Figure S14. A and **B.** Fluorescence spectra at different temperatures (from 25 to 95 °C, ΔT=10 °C) of DMAzo-TBA and DMAzo-T30695, respectively, excited at 255 nm acquired in the region 300-460 nm. **C**. Comparison between fluorescence spectra of DMAzo-TBA dissolved in 5KWE and 5KEW-TEA blends. **D**. Comparison between fluorescence spectra of DMAzo-T30695 dissolved in 5KWE and 5KEW-TEA blends.

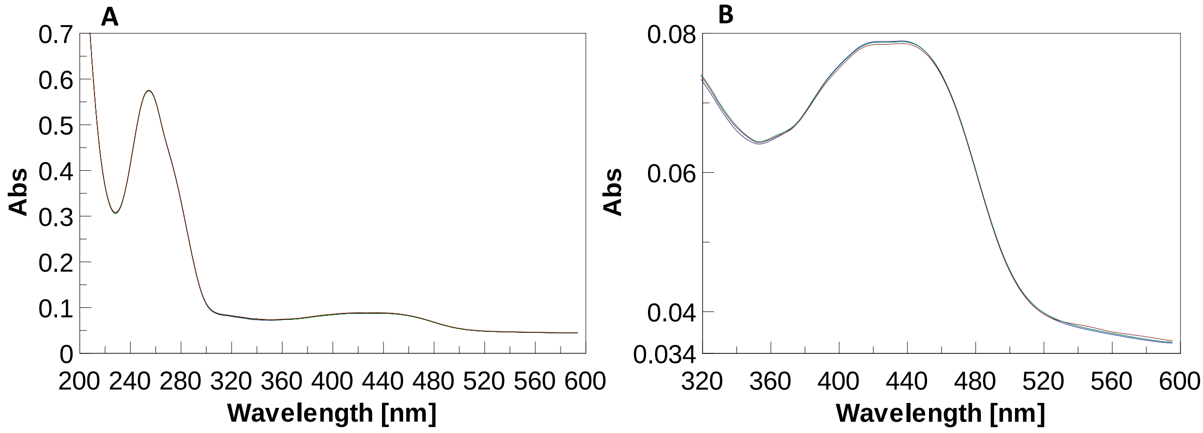

Figure S15. Time-dependent UV spectra of 5'-DMAzo-T30695 in 5KEW-TEA blend [ON] = 3.5x10^{-6}M after LED irradiation at 436 nm. Full spectra (**A**) and an enlargement between 300-600 nm (**B**) corresponding to the absorption band of the 5'-DMAzo moiety. No significant changes of the band intensities are detectable before and after the irradiation.

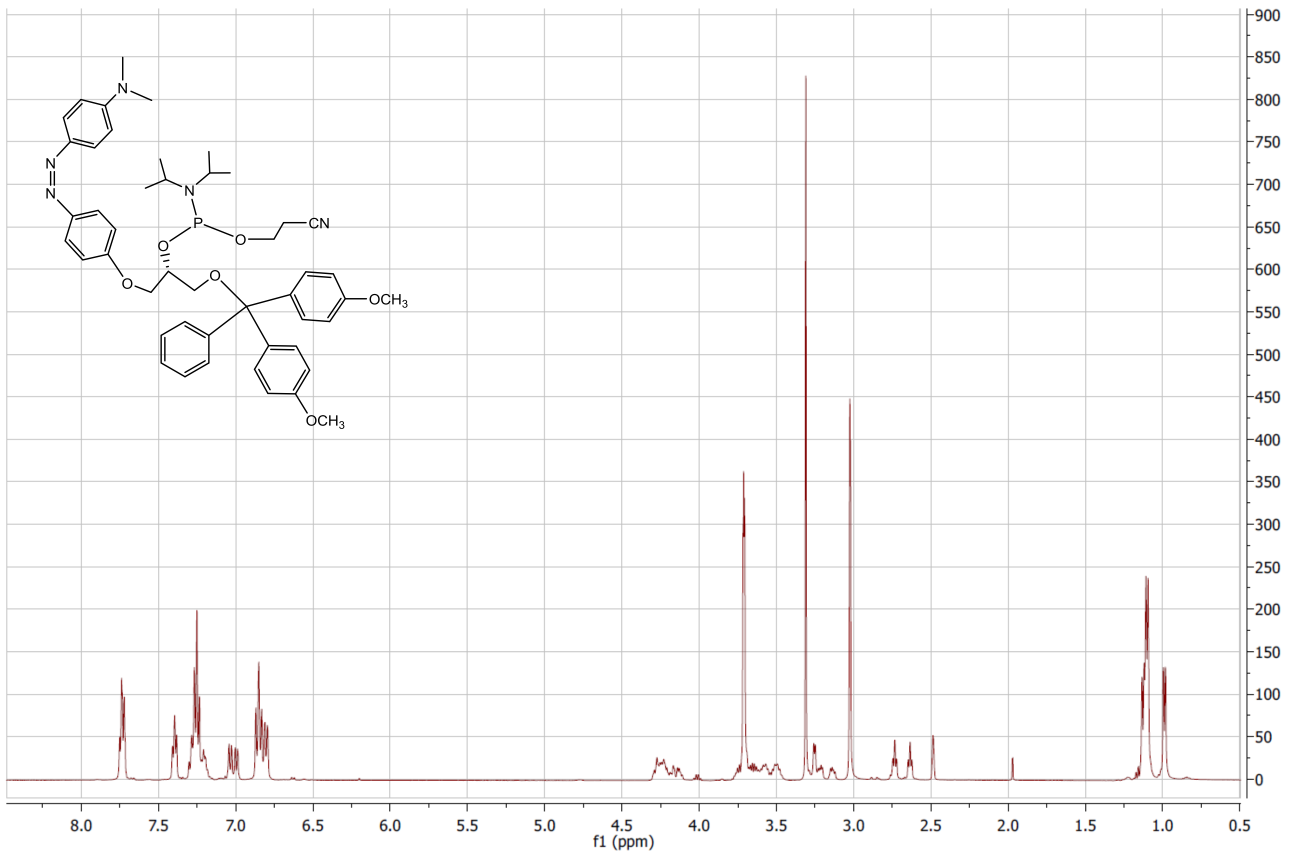

Figure S16. ^{1}H NMR spectrum of compound **7** (scheme 1 in the main text) in DMSO-d_6 performed by means of 500 MHz Varian.

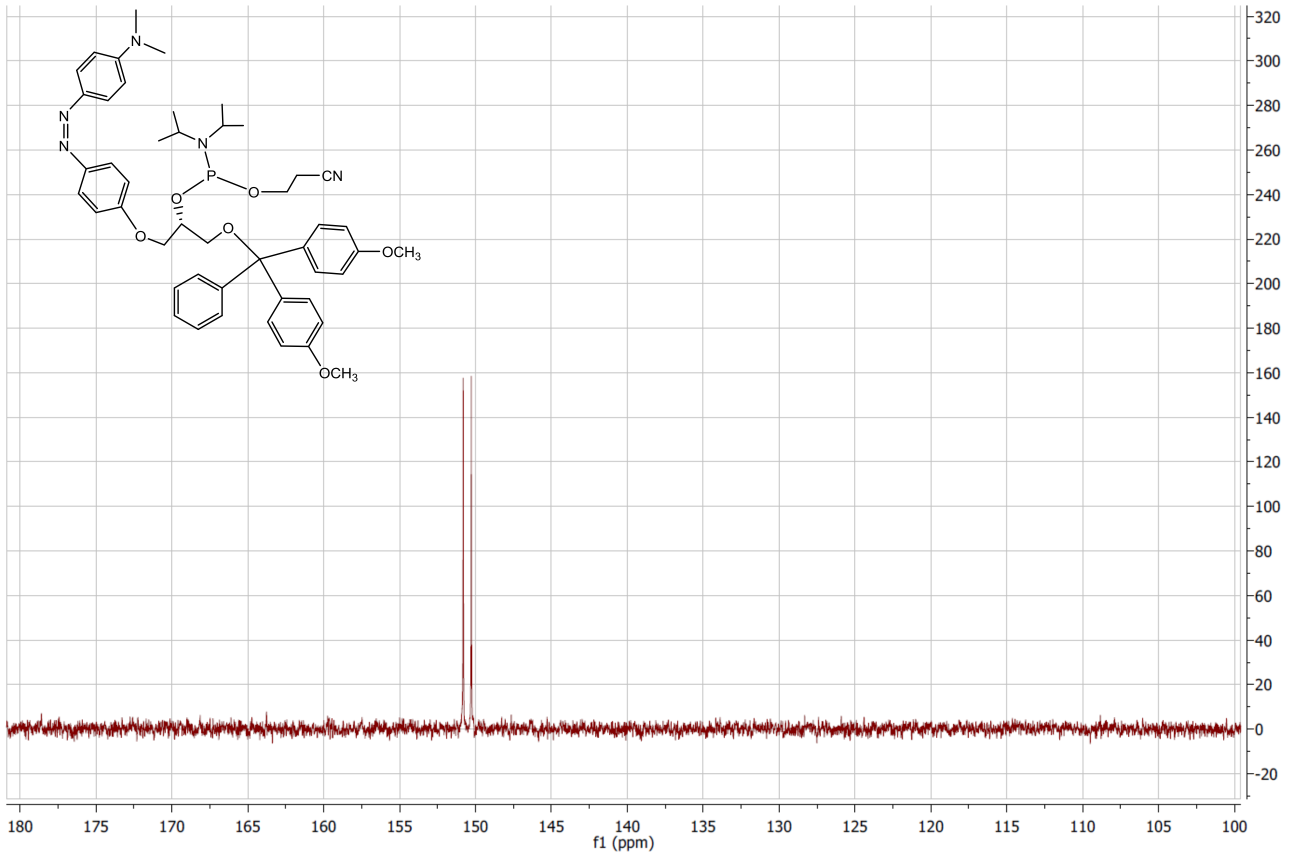

Figure S17. ^{31}P NMR spectrum of compound **7** (scheme 1 in the main text) in DMSO-d_6 performed by means of 500 MHz Varian.

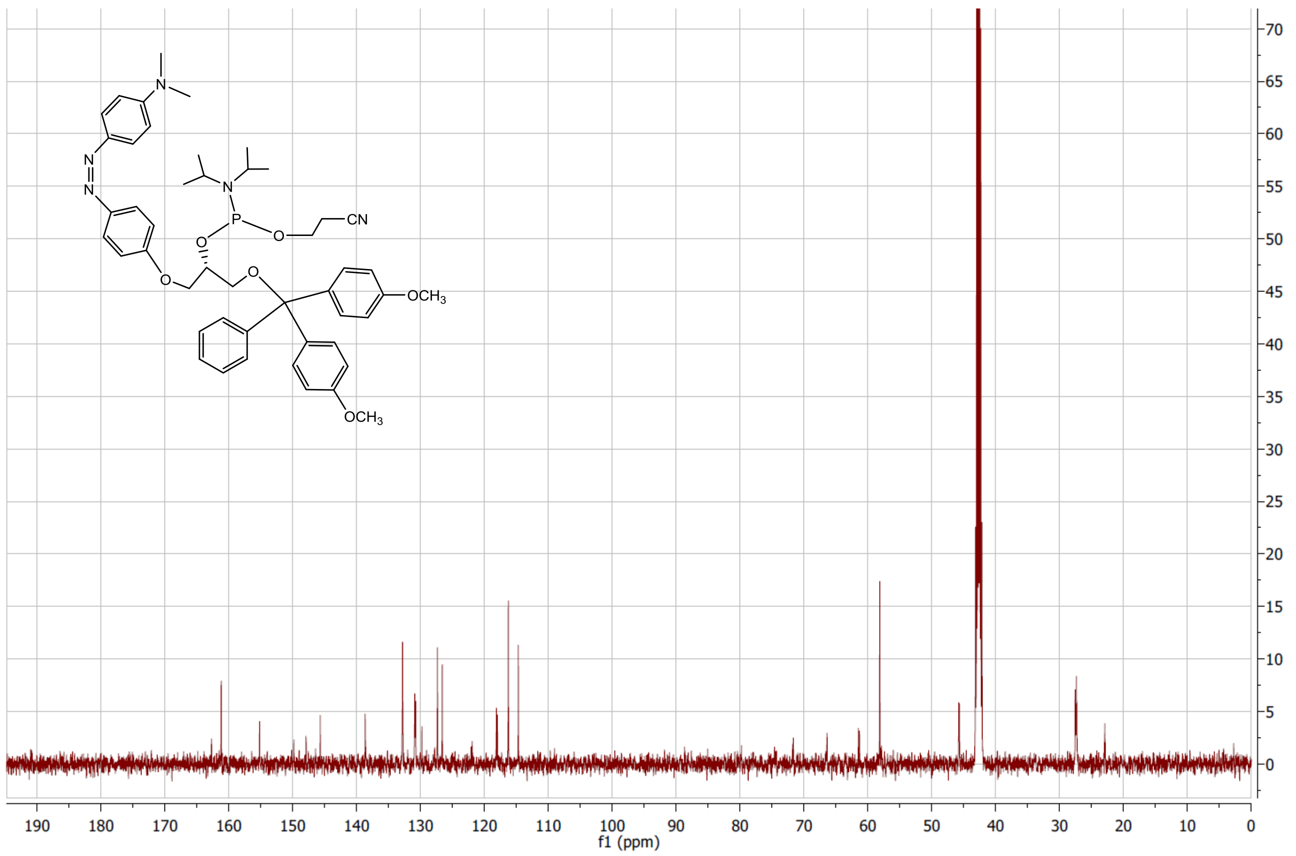

Figure S18. ^{13}C NMR spectrum of compound **7** (scheme 1 in the main text) in DMSO-d_6 at 125 MHz Varian.

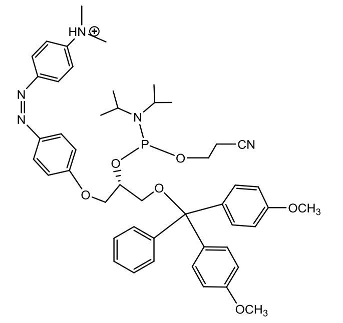

Chemical Formula: $C_{47}H_{57}N_5O_6P^+$
Exact Mass: 818,4041
Molecular Weight: 818,9752
m/z: 818.4041 (100.0%), 819.4075 (50.8%), 820.4108 (12.6%), 819.4011 (1.8%), 821.4142 (1.2%), 820.4083 (1.2%)
Elemental Analysis: C, 68.93; H, 7.02; N, 8.55; O, 11.72; P, 3.78

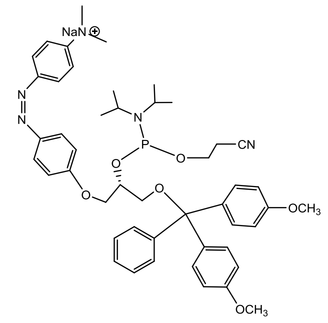

Chemical Formula: $C_{47}H_{56}N_5NaO_6P^+$
Exact Mass: 840,3860
Molecular Weight: 840,9570
m/z: 840.3860 (100.0%), 841.3894 (50.8%), 842.3928 (12.6%), 841.3831 (1.8%), 843.3961 (1.2%), 842.3903 (1.2%)
Elemental Analysis: C, 67.13; H, 6.71; N, 8.33; Na, 2.73; O, 11.41; P, 3.68

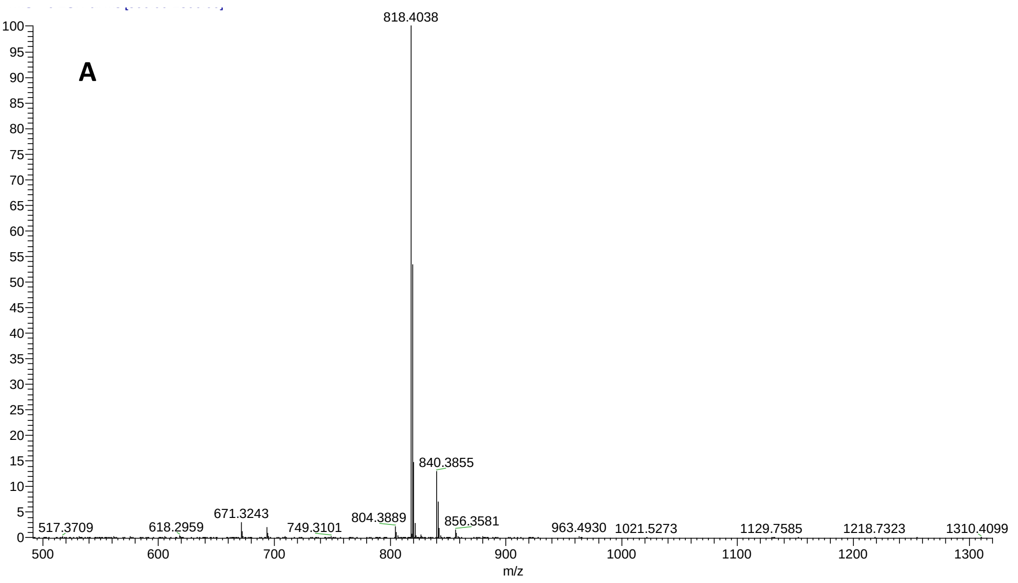

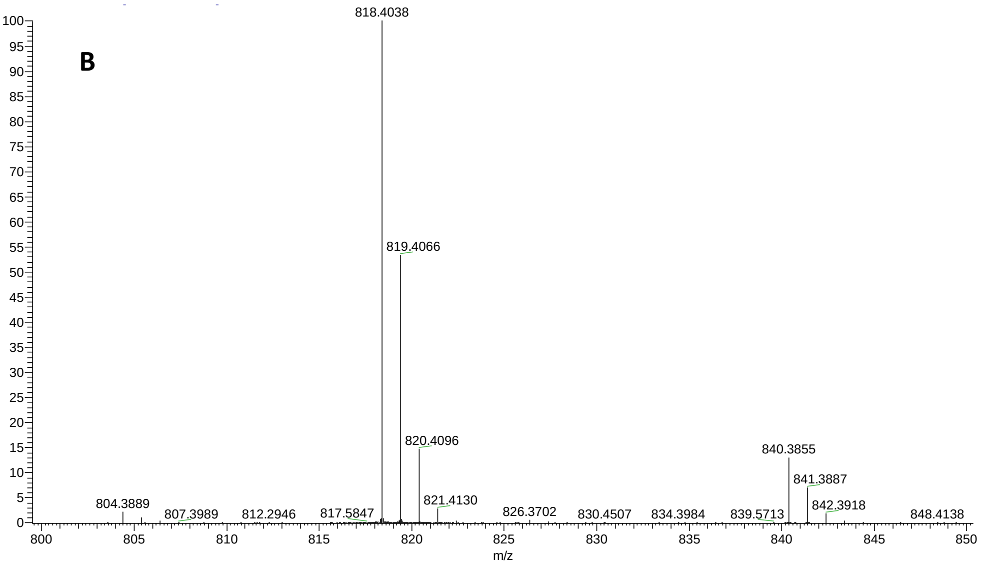

Figure S19. **A**. HRMS spectrum of compound **7** (scheme 1 in the main text) and **B.** enlargement of the m/z scale of the same spectrum. The theoretical mass value for the protonated and metalled (Na^+) mono-charged ions are reported in the upper pictures.
